# Deciphering the unique autoregulatory mechanisms and substrate specificity of the understudied DCLK3 kinase linked to neurodegenerative diseases

**DOI:** 10.1016/j.jbc.2025.110664

**Published:** 2025-09-01

**Authors:** Jason D. Lu, Peng Zhao, Anup Prasad, Neha Gupta, Nathan Gravel, Tej Shidhaye, Lance Wells, Samiksha Katiyar, Natarajan Kannan

**Affiliations:** 1 Department of Biochemistry and Molecular Biology, University of Georgia, Athens, Georgia, USA; 2 Complex Carbohydrate Research Center, University of Georgia, Athens, Georgia, USA; 3 Institute of Bioinformatics, University of Georgia, Athens, Georgia, USA

**Keywords:** cell signaling, bioinformatics, post-translational modification, drug discovery, kinase inhibitors, deep learning, proteomics, conformational changes, allosteric regulation

## Abstract

Protein kinases represent one of the largest and most druggable protein families. Despite considerable progress in their understanding, approximately one-third of human kinases remain poorly characterized, known as the "dark" kinome. Doublecortin-like kinase 3 (DCLK3), a member of this elusive group, has emerged for its involvement in neuroprotection in Huntington’s disease and other neurodegenerative disorders. Still, its cellular substrates and regulatory functions remain unknown, hindering progress in therapeutic intervention. Unlike its paralog, DCLK1, whose regulation involves a C-terminal segment docking into the ATP-binding pocket, DCLK3 lacks such a tail, suggesting divergent regulatory mechanisms. Through computational and experimental analyses, we discovered that DCLK3 autophosphorylates its truncated tail, tethering it to the catalytic domain in a manner distinct from DCLK1. Using a deep learning model trained on peptide-library datasets, we predicted Tau, a microtubule-associated protein, as a putative DCLK3 substrate, which we validated using *in vitro* assays and mass spectrometry. Additionally, DCLK3 exhibits a relatively fast turnover with a cellular half-life of approximately 15 h that can be rescued by MG132-mediated proteasomal inhibition, which results in DCLK3 polyubiquitination and cellular accumulation. Collectively, these results provide the first structural and functional insights into DCLK3, revealing a unique autoregulatory mechanism and a potential therapeutic target for neurodegenerative disorders.

Eukaryotic protein kinases are an essential class of enzymes that regulate many cellular processes by catalyzing the transfer of gamma phosphate from ATP (adenosine triphosphate) (1) to the hydroxyl group of a serine, threonine, or tyrosine in a protein or peptide substrate. The human kinome consists of more than 500 protein kinases. However, approximately one-third remains uncharacterized and is therefore classified as “dark” kinases (2). This knowledge gap in the dark kinome has limited the exploration of these kinases as therapeutic targets (3).

Within the ‘dark’ kinome is Doublecortin-like kinase 3 (DCLK3), which belongs to the Doublecortin family of kinases consisting of multiple paralogs (DCLK1 and DCLK2) and alternatively spliced isoforms (4, 5). Although DCLK1 has been extensively studied following its initial identification in developing mouse brain (6, 7), far less is known about DCLK3, with limited information regarding its structure, functions, and mechanisms. Moreover, the expression of DCLK3 at the transcript and protein levels is much lower relative to DCLK1 and DCLK2 across various cellular environments. This difference in expression and paucity of insights contributes to the gap in understanding between DCLK3 and its paralogs.

DCLK1 has been shown to orchestrate several neurodevelopmental processes (8), such as neurogenesis (9), neuronal migration (10), and apoptosis (11), and its elevated expression has been correlated with worse clinical outcomes in multiple cancer types (12, 13). Due to the clinical significance of DCLK1, several studies have sought to investigate the physiological relevance of DCLK3 (14, 15, 16, 17), associating it with neuroprotection in Huntington’s disease mice (14) and colorectal cancer progression (17). Additional studies knocking out DCLK3 have resulted in memory deficits along with transcriptomic changes associated with synaptic plasticity (15). Even so, the molecular mechanisms governing its role in neurons and cancer remain unknown.

At the protein domain level, DCLKs are composed of N-terminal doublecortin (DCX) domains, central kinase domains homologous to Ca^2+^/calmodulin-dependent kinases (CaMKs), and a variable C-terminal tail segment (5). In classical CaMKs, the kinase domain is auto-inhibited by the C-terminal tail at reduced Ca^2+^ levels (18, 19). At elevated Ca^2+^ levels, the kinase domain is activated by release of the auto-inhibitory tail (20). Much like CaMKs, DCLK1 is auto-inhibited by an extended C-terminal, which functions as a pseudosubstrate, sterically occluding the ATP-binding cleft and thereby suppressing catalytic activity (21). Unlike DCLK1 and DCLK2, DCLK3 is devoid of an extended auto-inhibitory tail, suggesting a divergent mechanism of regulation or lack thereof (5). Interestingly, none of the DCLK family members possess Calmodulin-binding motifs, indicating Calmodulin-independent regulation. However, some kinases within the CaMK family, such as PSKH1, which lack the Calmodulin-binding motif, are known to be allosterically regulated by Ca^2+^-sensing proteins (22).

Autophosphorylation illustrates another well-studied regulatory feature found in both CaMK and other protein kinases (23, 24, 25, 26, 27, 28). In CaMKII, autophosphorylation at residue T286, within the C-terminal Calmodulin-binding domain, facilitates Ca^2+^/calmodulin-independent activity by relieving C-terminal tail auto-inhibition (29). For DCLK1, autophosphorylation of the C-tail suppresses catalytic activity, ultimately inhibiting autophosphorylation of its microtubule-binding DCX domains (27). The status of autophosphorylation in DCLK3 and its impact on catalytic activity is yet to be determined.

Herein, we investigate the structural features and regulatory behavior of DCLK3 through post-translational modifications (PTMs)—particularly C-terminal autophosphorylation. Employing AlphaFold 3 (AF3) structural modeling in tandem with molecular dynamics (MD) simulations, we reveal that autophosphorylation stabilizes a conformation in which the DCLK3 C-tail engages within the substrate-binding groove in a manner distinct from auto-inhibition observed in DCLK1. This unique conformation is preserved by positively charged arginine residues in the αF-αG loop of the kinase domain. Utilizing Phosformer-ST (30, 31), a deep-learning transformer model designed to predict kinase-specific phosphorylation sites, we identify and experimentally confirm Tau, a microtubule-associated protein, to be directly phosphorylated by DCLK3—validating the first DCLK3 substrate *in vitro*. Furthermore, we explore DCLK3 regulation in cells, revealing it to be a relatively short-lived protein whose levels are controlled by the polyubiquitination of lysine residues. Indeed, treatment of HEK293T cells with the proteasome inhibitor MG132 results in the accumulation of polyubiquitinated DCLK3, which we confirm by mass spectrometry (MS). Collectively, our results provide the first molecular and mechanistic framework for further investigating the functional role of DCLK3 in normal and disease states.

## Results

### Structural divergence and cellular expression of DCLK3

**Figure 1:**
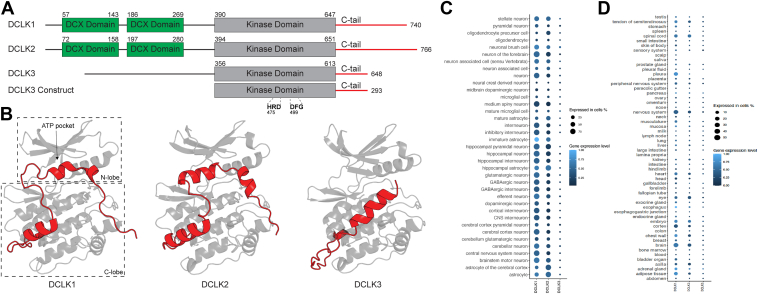
**Domain architecture and structural comparison of DCLK3 isoforms.***A*, domain schematic of the major isoforms for DCLK1, DCLK2, and DCLK3 shown alongside the truncated experimental DCLK3 construct designed and optimized for *E. coli* expression. Identical kinase domains and C-tails are shown in *grey* and *red*, respectively. DCLK1 and DCLK2 contain two doublecortin (DCX) domains shown in *green*. Key active site motifs (HRD and DFG) are labeled in the DCLK3 construct. *B*, structural representations of C-tail (*red*) interacting with the kinase domain in the three DCLK paralogs. A crystal structure is shown for DCLK1 (PDBID: 6KYQ). AlphaFold 3 models of the kinase domain and tail are shown for DCLK2 and DCLK3. *C*, dot plot showing gene expression in human brain for DCLK paralogs from CELLxGENE database. *D*, dot plot showing gene expression in other human tissues for DCLK paralogs. Percent of cells expressing the gene is represented as dot size and level of expression is represented by *blue* gradient.

The DCLK family consists of three closely related paralogs—DCLK1, DCLK2, and DCLK3—that give rise to multiple isoforms through alternative splicing (5). Figure 1*A* showcases the domain architecture of each major DCLK isoform. DCLK1 and DCLK2 bear two DCX domains (green), which are missing in the dominant isoform of DCLK3. Another pertinent difference is in the flanking C-terminal segment: DCLK1 and DCLK2 have extended tails measuring 93 and 115 amino acids in length, respectively, whereas DCLK3 has a much shorter 35-residue-long tail (Fig. 1*B*).

Due to the absence of experimentally derived structural data for DCLK2 and DCLK3, we deployed AlphaFold 3 (32) to generate structural models and evaluated the models using the predicted local distance difference test (pLDDT) score. Scores above 90 are labeled "very high" confidence; 70 to 90, "high" confidence; 50 to 70, "low” confidence; and below 50, "very low” confidence. While the kinase domains of DCLK2 and DCLK3 exhibit a high degree of confidence, their C-terminal tails were predicted with a much lower confidence score (Fig. S1), reflecting the predicted intrinsic disorder of these regions (Fig. S2) (33).

Comparison of the kinase domains and adjacent tails shows distinct binding modalities among the paralogs (Fig. 1*B*). DCLK1 (PDBID: 6KYQ) and DCLK2 (AF3 model) display C-tails that occlude ATP binding by occupying the ATP binding pocket and extend to the substrate binding regions. In contrast, the compact C-tail of DCLK3 (AF3 model) lacks the length to extend across the ATP-binding site but partially occludes the substrate binding C-lobe (Fig. 1*B*).

To delineate the individual expression patterns of DCLK paralogs, we analyzed single-cell RNA-sequencing data encompassing nearly 18 million individual brain cells, available through the CELLxGENE repository (34). Our analysis revealed DCLK3 transcript levels considerably lower compared to its paralogs, DCLK1 and DCLK2 (Fig. 1, *C* and *D*). While DCLK1 and DCLK2 are expressed robustly in neuronal cell types as well as in other tissue types in the human body (Fig. 1*D*) (4, 5), DCLK3 is expressed at much lower levels in various neuronal cell types and tissue types, suggesting a unique mode of cellular regulation. The undetectable levels of DCLK3 expression also provide a plausible explanation for why DCLK3 has eluded experimental characterization.

### Biochemical characterization of DCLK3 reveals autophosphorylation

Autophosphorylation is a common mode of regulation in protein kinases (24, 25, 26, 27, 28). To determine whether DCLK3 is autophosphorylated, we used complementary kinase activity assays, including both *in vitro* phosphorylation and ADP-Glo bioluminescence-based assays.

**Figure 2:**
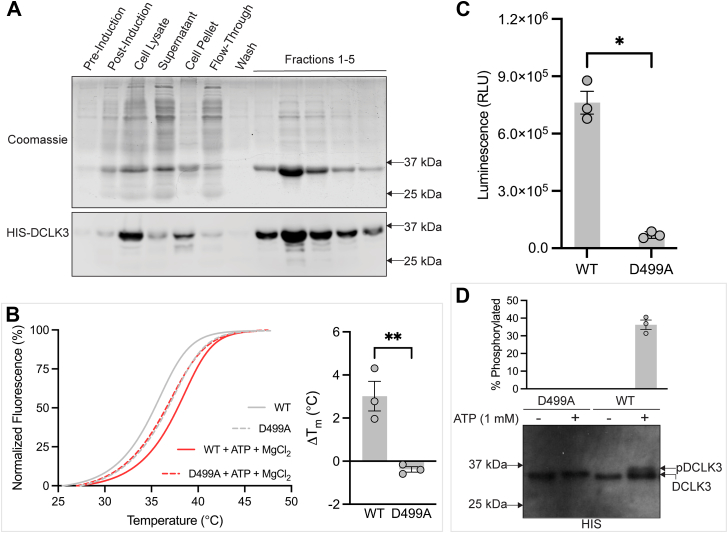
**DCLK3 kinase activity and autophosphorylation.***A*, SDS-PAGE and Western blot analysis of DCLK3 expression and purification. SDS-PAGE gel stained with Coomassie (*top*) and immunoblot (*bottom*) show expression and purification of DCLK3 after immobilized metal affinity chromatography (IMAC). Immunoblotting was performed using anti-HIS antibody. *B*, differential scanning fluorimetry assay of DCLK3 stability. Normalized denaturation curves for DCLK3 WT and the catalytically inactive DFG mutant D499A, in the presence or absence of ATP and MgCl_2_ (*left*). WT shows a Mg^2+^-ATP-dependent shift, but no change is observed in D499A. Quantification of the change in melting temperature (ΔTm) for WT and D499A DCLK3 in the presence or absence of ATP and MgCl_2_ (*right*). Data is shown as the mean ± SEM for three replicate experiments. Statistical analysis was performed using a two-tailed unpaired *t* test (*p* = 0.0083). *C*, luminescence-based ADP-Glo kinase assay of WT and D499A (kinase dead) DCLK3 autophosphorylation. The D499A mutation results in a significant decrease in kinase activity (*p* < 0.05). Data is shown as mean luminescence ± SEM. Statistical analysis was performed using a two-tailed unpaired *t* test (*p* = 0.000333). *D*, western blot analysis of WT and D499A (kinase dead) DCLK3 kinase assay. Autophosphorylated DCLK3 (pDCLK3) is detected by a size shift present in WT but not D499A. Immunoblotted with anti-HIS antibody.

Our cell-free assays used a truncated DCLK3 construct encoding only the kinase domain and the C-tail (residues 356–648, Fig. 1*A*). Wild-type (WT) and catalytically inactive (kinase-dead, KD; D499A mutant) versions of this construct were expressed in *E. coli* and purified (Fig. 2*A*). The construct was verified *via* SDS-PAGE and immunoblotting, making it the first successful purification of recombinant DCLK3 in *E. coli*. A detailed purification profile is shown in Fig. S3.

We next employed differential scanning fluorimetry (DSF) to evaluate the ability of our recombinant constructs to bind ATP and MgCl_2_. WT-DCLK3 displayed a marked increase in thermal stability upon addition of ATP and MgCl_2_, indicative of ligand binding. In contrast, the kinase-dead mutant (D499A) showed no such thermal shift, consistent with a canonical kinase mechanism of Mg^2+^-ATP coordination *via* the DFG-Aspartate (D499) (Fig. 2*B*) (35). To assess the necessity of Mg^2+^ for ATP binding, we repeated the assay in the absence of Mg^2+^. Under these conditions, ATP alone failed to induce any significant change in the thermal denaturation profile, suggesting that Mg^2+^ is essential for ATP binding (Fig. S4).

To further examine the autophosphorylation of WT- and KD-DCLK3, we conducted ADP-Glo assays, which measure kinase activity by quantifying the production of ADP (adenosine diphosphate) as a luminescence signal. WT-DCLK3 exhibited significantly higher relative luminescence units (RLU) compared to KD-DCLK3 (*p* < 0.05), even in the absence of an exogenous substrate (Fig. 2*C*). These data are consistent with autophosphorylation and confirm DCLK3 as a catalytically active kinase.

To substantiate these findings, we performed *in vitro* kinase assays to detect an electrophoretic mobility shift indicative of phosphorylation (36). WT- and KD-DCLK3 were incubated in kinase reaction buffer in the presence or absence of ATP. As shown in Figure 2*D*, ATP treatment led to a molecular weight shift in WT-DCLK3 (pDCLK3) but not in the kinase dead (KD-DCLK3) control. Quantification of this shift in WT-DCLK3 across three biological replicates showed a mean phosphorylation of 36.25%.

### DCLK3 autophosphorylation stabilizes its C-terminal segment

**Figure 3:**
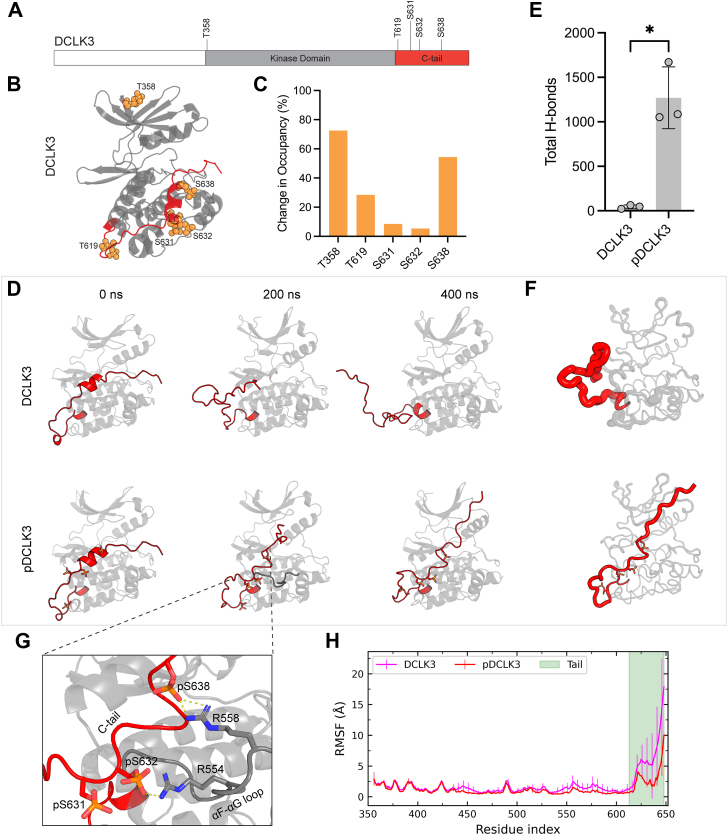
**DCLK3 autophosphorylation and C-tail regulation.***A*, mapping of DCLK3 autophosphorylation sites. Domain schematic of DCLK3 with the DCLK3 construct used colored in *grey* and *red*. Individual phosphorylation sites are labeled above. *B*, AlphaFold 3 model of DCLK3 showing phosphorylated residues (*orange*). *C*, percent change in occupancy of key DCLK3 autophosphorylation sites detected by LC-MS/MS (*bottom right*). Samples used for LC-MS/MS are shown in Fig. S5. *D*, snapshots at different time points of the MD simulations show the effect of phosphorylation on tail dynamics (*red*). The unphosphorylated tail interacts weakly with the kinase domain at initial stage and detaches at 200 ns. In contrast, the tail in phosphorylated DCLK3 stably interacts with kinase domain and remains tethered to the substrate-binding lobe throughout the trajectories. *E*, the count of total H-bonds observed between serine residues (S632, S638) and arginine residues (R554, R558) in the phosphorylated and unphosphorylated DCLK3 simulations. Statistical analysis was performed using a two-tailed unpaired *t* test (*p* = 0.0037). *F*, average structure of DCLK3 and pDCLK3 simulations are shown in the putty representation where the mean RMSF values were used as the B-factor. *G*, the zoomed-in snapshot of pDCLK3 shows the H-bonds between phosphate groups (pS632, pS638) and arginine residues (R554, R558) which tethered the tail to the substrate-binding lobe. *H*, the averaged RMSF of DCLK3 and pDCLK3 simulations.

To expand upon DCLK3 autophosphorylation, we performed LC-MS/MS analysis of DCLK3 before and after ATP treatment (Fig. S5; top row). This analysis confirmed DCLK3 autophosphorylation of 5 S/T residues (T358, T619, S631, S632, and S638) at 120 min, while negligible phosphorylation was detected in KD-DCLK3 control or at 0 min (Fig. 3*A*). The autophosphorylation sites were mapped onto the DCLK3 structural model (Fig. 3*B*), and the change in occupancy was quantified (Fig. 3*C*). Given that the majority of the autophosphorylation sites occurred on the C-terminal segment of DCLK3, we next explored potential regulatory features of these modifications through computational methods.

To investigate the structural dynamics of DCLK3 autophosphorylation, we generated structural ensembles of both unphosphorylated (DCLK3) and phosphorylated (pDCLK3) DCLK3 using AlphaFold 3. Conformational clustering based on the root mean square deviation (RMSD), revealed three distinct clusters for both unphosphorylated and phosphorylated forms. Among the unphosphorylated models, the most populated conformation (39.60% of the total population) showed the C-tail positioned along the substrate binding groove of the kinase domain (Fig. S6). When modeled with the autophosphorylated residues, there was a shift in the population predicted in the tail-bound conformation (61.20%), suggesting that autophosphorylation confines the conformational heterogeneity of the C-terminal tail.

To further understand the role of the C-terminal tail segment on structural stability and dynamics, we performed MD simulations on a representative structure of the major conformer of DCLK3 and pDCLK3 (Fig. 3*D*). Root mean square fluctuation (RMSF) of backbone atoms revealed decreased flexibility of the C-tail in pDCLK3 due to coordination of the negatively charged phosphate groups in the tail by positively charged arginine residues in the kinase domain (Fig. 3, *F* and *G*). Particularly, the hydrogen bonds between phosphorylated residues in the C-tail (pS632 and pS638) and positively charged residues in the kinase domain (R554 and R558) were consistently observed throughout the trajectories across multiple replicates (Fig. 3*E*). These interactions tether the C-tail to substrate binding regions of the kinase domain (αD-helix and the αF-αG loop). This mode of interaction is distinct from DCLK1, where the C-tail extends into the ATP-binding pocket (5).

### Tau is a putative DCLK3 substrate *in vitro*

To identify potential substrates of DCLK3, we utilized Phosformer-ST (30, 31), a transformer-based model trained to predict peptide substrates based on experimentally derived peptide-library kinase screens (23) and the evolutionary, structural, and functional features encoded in primary sequences. Using the catalytic domain of DCLK3 (residues 356–613) as the input and a dataset of known phosphorylated peptides from the human proteome (23, 37), Phosformer-ST identified high-confidence phosphorylation sites with scores greater than 0.98 in several microtubule-associated proteins, including Tau (Fig. S7 and Table S1).

**Figure 4:**
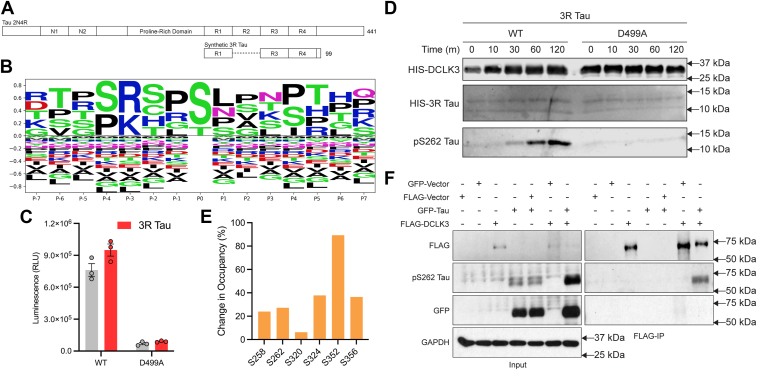
**Phosformer-ST based prediction and *in vitro* validation of DCLK3 and Tau.***A*, schematic representation of 2N4R Tau and experimental synthetic 3R Tau construct. *B*, sequence logo of the Phosformer-ST consensus of DCLK1/2/3 substrate preference generated by predicting on all viable phospho-sites found in Tau with a cutoff of (>0.5) for the associated Phosformer-ST score. Sequence preference is shown indicating the preferred amino-acids (positive Y-axis) and non-preferred amino acids (negative Y-axis) for the 15-mer sequence. *C*, luminescence-based ADP-Glo kinase assay of WT and D499A (kinase dead) DCLK3 in the presence and absence of 3R Tau. Data is shown as mean luminescence ± SEM. *D*, Western blot analysis of time-course kinase assay showing phosphorylation of S262 in 3R Tau by WT and D499A (kinase dead) DCLK3. *Top* and *middle panels* show DCLK3 and 3R Tau levels, respectively. The *bottom panel* shows phosphorylated 3R Tau at S262, observed with WT but not D499A. Immunoblotted with anti-HIS and anti-pS262 Tau antibodies. *E*, percent change in occupancy of key 3R Tau phosphorylation sites detected by LC-MS/MS. *F*, co-immunoprecipitation of DCLK3 and Tau. HEK293T cells were either transfected with GFP-Vector, FLAG-Vector, FLAG-DCLK3, or GFP-Tau alone or co-transfected as indicated. Western blot analysis was performed on whole cell lysates (input) and FLAG immunoprecipitates (FLAG-IP). GAPDH is shown as a loading control. Immunoblotting was performed with anti-FLAG, anti-GFP, anti-pS262 Tau, and anti-GAPDH antibodies.

To further investigate Tau as a potential substrate, Phosformer-ST was employed to systematically score each serine (S) and threonine (T) residue within the 2N4R Tau isoform (Fig. 4*A*). A ranked list of all 15-mer peptides in 2N4R Tau, categorized by Phosformer-ST score for DCLK3-mediated phosphorylation, is shown in Table S2, alongside scores for the closely related DCLK1 and DCLK2 paralogs. Notably, all the high-scoring peptides for DCLK1, DCLK2, and DCLK3 (score > 0.5) display Tau consensus motif features, including a preference for serine or proline at the −4 position and arginine or lysine at the −3 position (Fig. 4*B* and Table S2). These findings suggest that DCLK family kinases exhibit specificity for certain residues and sequence motifs in Tau, thereby guiding phosphorylation events critical for regulatory function.

To experimentally validate Tau as a DCLK3 substrate, we utilized a synthetic 3R Tau construct designed for expression in a cysteine-free (K19CF) expression vector, incorporating an N-terminal histidine-tag for purification (38). Protein sequence, domain architecture, and purification profile of synthetic 3R Tau are shown in Fig. S8. For ease of annotation, phosphorylation sites on 3R Tau were mapped using the 2N4R Tau numbering scheme (Table S2).

Initial validation efforts employed the bioluminescence-based ADP-Glo assay. While the average relative luminescence units (RLU) indicated marginal ADP production, this difference was not statistically significant (*p* > 0.05), likely due to background autophosphorylation (Fig. 4*C*). Importantly, the catalytically inactive KD-DCLK3 construct confirmed that no autophosphorylation or phosphorylation of synthetic 3R Tau occurred.

To directly assess the phosphorylation of synthetic 3R Tau by DCLK3, we performed an *in vitro* kinase assay in the presence of ATP/Mg^2+^ over a 2-h period. The assay revealed detectable 3R Tau phosphorylation at S262 beginning at 30 min, with levels increasing until the reaction was quenched at 120 min (Fig. 4*D*). Phosphorylation was validated through immunoblotting using a pS262-specific Tau antibody. Total protein levels for both DCLK3 and Tau were verified using HIS-antibody, with no phosphorylation detected in the KD-DCLK3 control. Complete immunoblot data, including controls, are provided in Fig. S9.

To map the sites of phosphorylation, protein samples from the *in vitro* kinase assay were resolved *via* SDS-PAGE and stained with Coomassie blue. Protein bands corresponding to 3R Tau were excised and subjected to MS analysis (Fig. S5; red box, bottom row). This analysis identified several phosphorylation sites on serine/threonine residues within the synthetic 3R Tau construct. Notably, the major phosphorylation sites—S352, S324 and S356—are localized within KXGS motifs (Figs. 4*E* and S8), which are known to mediate Tau-microtubule interaction and have been implicated in tauopathies (39, 40). Among these, S324 was corroborated by Phosformer-ST-predictions, achieving a higher confidence score of 0.764, supporting the model’s zero-shot prediction capabilities (Fig. S8*B* and Table S2). Although the other phosphorylation sites (S258, S320, S352, S356) exhibited lower scores (<0.5), they rank within the top percentile of all 15-mer Tau peptides and contain Tau consensus motif features such as an arginine or lysine at the −3 position. Collectively, these studies confirm Tau as a *bona fide* DCLK3 substrate *in vitro* and map the specific phosphorylation events at functionally relevant motifs in Tau regulation.

To further investigate the physical interaction between DCLK3 and Tau in mammalian cells, we conducted co-immunoprecipitation assays. HEK293T cells were co-transfected with plasmids expressing FLAG-DCLK3 and GFP-Tau, followed by immunoprecipitation using FLAG-agarose beads. Immunoprecipitated proteins were resolved on SDS-PAGE and immunoblotted with FLAG, GFP, and p262Tau-specific antibodies. Phosphorylated Tau (pS262) co-immunoprecipitated with FLAG-DCLK3, whereas unphosphorylated Tau did not (Fig. 4*F*), suggesting that DCLK3 preferentially interacts with the phosphorylated form of Tau in cells. Full-length immunoblots, including controls, are shown in Fig. S10.

### DCLK3 undergoes ubiquitin-mediated proteasomal degradation

**Figure 5:**
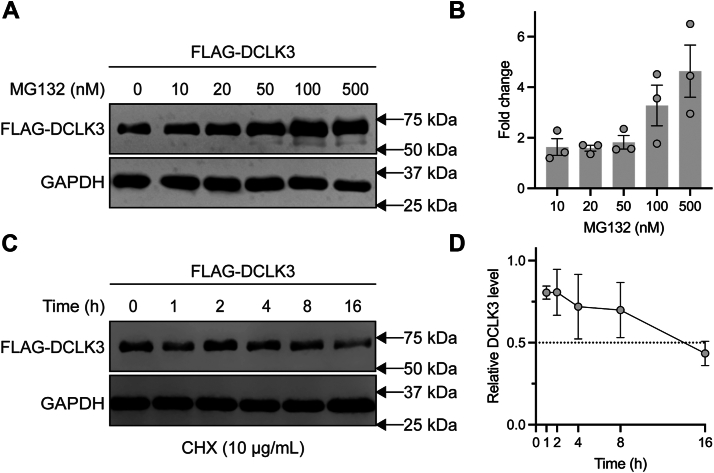
**DCLK3 proteasome-mediated degradation, and half-life.***A*, Western blot analysis of FLAG-DCLK3 in HEK293T cells with increasing MG132 proteasome inhibitor treatment (0–500 nM) after 16 h and immunoblotted with anti-FLAG and anti-GAPDH antibodies. GAPDH is shown as a loading control. *B*, quantified fold change of FLAG-DCLK3 levels from *panel* (*A*) normalized to GAPDH. Data shown as mean values with SEM over three replicate experiments. *C*, Western blot analysis of FLAG-DCLK3 in HEK293T cells treated with 10 μg/ml cycloheximide (CHX) over the course of 16 h. GAPDH is shown as a loading control. *D*, quantification of FLAG-DCLK3 levels from *panel* (*C*) shown as mean value and SEM over three replicate experiments.

Because DCLK3 transcript levels are significantly reduced compared to its paralogs across various cell and tissue types (Fig. S11), we investigated whether DCLK3 is modulated by proteasomal degradation triggered by ubiquitination (41, 42). To uncover proteasomal involvement, we analyzed DCLK3 accumulation following treatment with MG132, a competitive inhibitor of the proteasome complex (41). HEK293T cells were transfected with FLAG-Vector or FLAG-DCLK3, 48 h post-transfection, cells were treated with MG132 at the indicated concentrations for 16 h. Cells were lysed in RIPA buffer, and lysates were resolved on SDS-PAGE and immunoblotted with FLAG and GAPDH antibodies to assess DCLK3 and total protein levels. Indeed, the accumulation of DCLK3 occurs upon inhibition of the proteasome in a concentration-dependent manner (Fig. 5*A*), and, when quantified, the highest treatment (500 nM) showed an approximately threefold increase compared to baseline (0 nM) (Fig. 5*B*). FLAG-Vector and GAPDH controls are shown in Fig. S12.

Next, we evaluated DCLK3 turnover using a cycloheximide chase assay, which blocks new protein synthesis by inhibiting translational elongation, allowing for the assessment of protein degradation rates (43). To understand this phenomenon, HEK293T cells were transfected with FLAG-Vector or FLAG-DCLK3. 48 h post-transfection, cells were treated with 10 μg/ml cycloheximide and harvested at the indicated time points. Cells were lysed in RIPA buffer, and lysates were resolved on SDS-PAGE and immunoblotted with FLAG and GAPDH antibodies to assess DCLK3 and total protein levels. As shown in Figure 5*C*, DCLK3 levels gradually decreased over the 16-h time course, whereas the GAPDH control remained constant. Quantification estimated a half-life of approximately 15.5 h (Fig. 5*D*). Full immunoblot data, including FLAG-vector and GAPDH controls, are shown in Fig. S12. These results suggest that DCLK3 degradation is mediated by the proteasome, which may explain its low cellular abundance. However, as these experiments utilized overexpressed DCLK3, the results may differ from endogenous protein.

**Figure 6:**
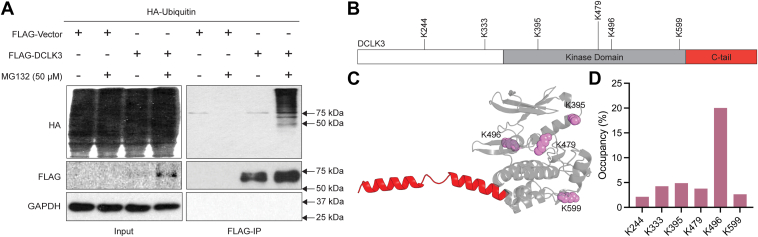
**DCLK3 ubiquitination.***A*, HEK293T cells were co-transfected with HA-Ubiquitin and either FLAG-Vector or FLAG-DCLK3. Post transfection (48 h) cells were treated with MG132 (50 uM) for 4 h. Whole-cell lysates (Input) and FLAG immunoprecipitates (FLAG-IP) were analyzed by Western blot with anti-FLAG, anti-HA, and anti-GAPDH antibodies. *B*, mapping of DCLK3 ubiquitination sites. Domain schematic of DCLK3 showing key ubiquitination sites. *C*, alphaFold 3 model of DCLK3 with corresponding ubiquitinated lysine residues (*purple*). N-terminal domain along with K244 and K333 are not shown due to low confidence (Fig. S1). *D*, percent occupancy of DCLK3 ubiquitination sites detected by LC-MS/MS. Samples collected for LC-MS/MS analysis are shown in Fig. S14.

Given that MG132 treatment led to DCLK3 accumulation, we next investigated whether DCLK3 undergoes ubiquitination, a crucial step in the proteasomal degradation pathway (42). To examine this, HEK293T cells were co-transfected with FLAG-DCLK3 and HA-Ubiquitin, 48 h post transfection cells were treated with 50 uM MG132 for 4 h, DMSO treatment was used as control. Cells were lysed in RIPA buffer and subjected to immunoprecipitation with FLAG-agarose beads. When immunoprecipitated with FLAG-agarose beads, higher molecular weight bands appeared suggesting the presence of polyubiquitin on DCLK3 (Fig. 6*A*). Full-length immunoblots, including ubiquitination and loading controls, are presented in Fig. S13.

To map specific ubiquitination sites, we performed MS on samples from HEK293T cells co-transfected with FLAG-DCLK3 and HA-Ubiquitin and treated with MG132. Cells were lysed in RIPA buffer and immunoprecipitated with FLAG-agarose beads. Total cell lysates and immunoprecipitated samples were resolved on SDS-PAGE and stained with Coomassie blue. Protein bands corresponding to 75 kDa (possibly FLAG-DCLK3) and higher molecular weight 75 to 250 kDa (possibly ubiquitinated FLAG-DCLK3) were cut and processed for MS analysis (Fig. S14, red box). Additionally, samples processed for MS analysis were also checked by immunoblotting with FLAG and HA antibodies to ensure the expression and ubiquitination of DCLK3 (Fig. S14). MS analysis revealed six lysine residues to be ubiquitinated: K244, K333, K395, K479, K496, and K599 (Fig. 6*B*). Four of these lysine residues (K395, K479, K496, and K599) reside within the catalytic domain, while the remaining two are in the N-terminal region. All six lysine residues are solvent-exposed based on the AlphaFold 3 model (Fig. 6*C*), and K496, located on the β8 strand in the kinase C-lobe, exhibited the greatest occupancy (Fig. 6*D*).

## Discussion

Since its discovery in mice, the elusive dark kinase, DCLK3, has been the least studied within the DCLK family (4). Several factors have stymied research surrounding DCLK3, including challenges expressing recombinant full-length DCLK3—likely due to its extensive, disordered N-terminal region (Fig. S2)—as well as its low cellular abundance and rapid turnover (Figs. 1*C* and S11). To overcome these constraints, we employed a bioinformatic-guided *in silico* approach to elucidate the structural features of DCLK3. Computational modeling was reinforced through the successful expression and purification of a truncated construct (Fig. 1*A*), allowing for cell-free *in vitro* experimentation. Finally, we utilized a mammalian cell expression system along with MS analysis to further corroborate our findings.

**Figure 7:**
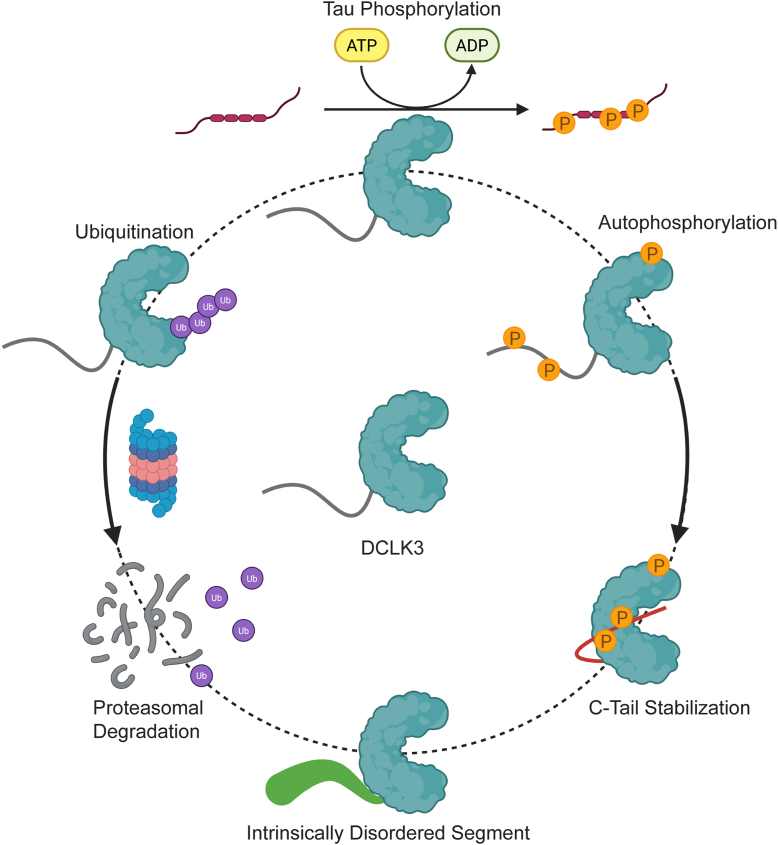
**DCLK3 multiple functions and modifications.** Schematic representation of DCLK3 features, functions, and regulatory modalities. Tau identified as a putative substrate of DCLK3 *in vitro* (*top*). Intrinsically disordered segment (IDS) of the DCLK3 C-tail shown in *green* with B-factor putty representation (*bottom*). DCLK3 undergoes ubiquitination, leading to proteasome-mediated degradation (*left*). Autophosphorylation of DCLK3 leads to stabilization of the C-tail along the substrate binding groove shown in *red* (*right*).

Here, we present the first exploration of DCLK3 at the structural and mechanistic level, identifying multiple modes of regulation, including a novel regulatory mechanism mediated through autophosphorylation (Fig. 7). Unlike the auto-inhibitory tails of DCLK1 and DCLK2, the C-tail of DCLK3 is considerably shorter, lacking the length to occlude the ATP-binding site like DCLK1 (Fig. 1*B*). Instead, structural modeling and molecular simulations indicate autophosphorylation stabilizes DCLK3 in a unique conformation with the C-tail bound along the substrate-binding groove, between the αD and αG helices in the C-lobe (Fig. 3*F*). An in-depth exploration shows paralog-specific arginine residues, spanning the αF-αG loop, coordinating with phosphorylated serine residues on the C-tail (Fig. 3*G*). Indeed, our AF3-generated DCLK3 structural ensemble showed a preference for the tail-bound conformation when the sites were phosphorylated (Fig. S6).

In addition to autophosphorylation and structural characterization, our observations expand upon the regulatory mechanisms governing the cellular abundance of DCLK3 (Fig. 7). Our findings indicate that it is readily ubiquitinated in HEK293T cells (Fig. 6*A*). In the typical cellular context, ubiquitination destines proteins for the proteasome-degradation pathway (41, 42). Evidently, proteasomal inhibition led to an accumulation of polyubiquitinated DCLK3, confirming the involvement of the proteosome in its regulation (Fig. 5*A*). We approximate a relatively short half-life of 15.5 h (Fig. 5*D*), although this measurement was acquired using overexpressed, not endogenous, DCLK3 in HEK293T cells. Notably, some of the ubiquitination sites, such as K479 and K496, are sequence adjacent to catalytically important HRD and DFG motifs, respectively (28). The highest occupancy K496 site is also adjacent to a VRP motif in the activation loop that can serve as a recognition motif for Siah family ubiquitin ligases (44). It is likely that ubiquitination of these sites could prevent substrate binding and catalysis in DCLK3. Reciprocally, the tail-bound conformation could block the ligation of ubiquitin onto these sites. The quantification of the solvent accessible surface area shows a decrease in solvent accessibility of K479 upon C-terminal phosphorylation (Fig. S15), suggesting potential crosstalk between C-tail phosphorylation and ubiquitination (45).

Furthermore, we provide the first identification and validation of Tau as a putative substrate of DCLK3 (Fig. 7). Tau is involved in neuronal microtubule dynamics and implicated in many neurodegenerative tauopathies such as Alzheimer’s disease (AD) (46). In our study, we demonstrate that DCLK3 directly phosphorylates Tau on S262, S324, and S356 residues, within the KXGS motifs, as confirmed by our Western blot and MS analysis. Aberrant hyperphosphorylation at these sites contributes to the destabilization of Tau-microtubule interactions (40), dissociation of Tau from microtubules (39), and formation of neurofibrillary tangles (47). Evidently, hyperphosphorylated Tau at S262 and S356 is observed in Alzheimer's disease brain (47). Thus, DCLK3-mediated Tau phosphorylation *in vitro* suggests a potential role in the pathogenesis of tauopathies. Moreover, it is possible that the subcellular localization of Tau phosphorylation is mediated by the microtubule association of both proteins. Although the major isoform of DCLK3 lacks the microtubule-associated DCX domain (Fig. 1*A*), it is present in a longer isoform (Ensembl transcript ID: ENST00000636136.2) (48).

Altogether, our work moves the structural understanding of DCLK3 forward and begins to clarify its nascent role in neurodegenerative disease, as well as in a subset of cancer types such as testicular cancer, where DCLK3 is differentially expressed in tumors relative to normal samples (Fig. S16). We reveal paralog-specific novel regulatory mechanisms in which kinase stability and abundance are licensed by autophosphorylation and ubiquitination. Furthermore, we highlight the microtubule-associated Tau as a novel DCLK3 substrate *in vitro*, underscoring its potential role in Tau-driven neurodegenerative diseases. This kinase-substrate interaction awaits confirmation *in vivo* and provides a rationale for exploring how the unique regulatory mechanisms of DCLK3 may be harnessed for targeted therapies.

## Experimental procedures

### DCLK3 expression and purification

The DCLK3 kinase domain (residues 356–648) was cloned into pET-28a(+) with a hexahistidine tag at the N-terminus by GenScript. The construct was co-transformed with lambda phosphatase into BL21 (DE3) cells to produce unphosphorylated DCLK3. A single colony was cultured in LB media, then diluted 1:100 into 1 L LB media. Cells were grown at 37 °C until reaching an OD of 0.6, then induced with 0.5 mM IPTG at 16 °C for 18 h. After harvesting, cells were resuspended in binding buffer (50 mM HEPES, 150 mM NaCl, 15% glycerol, pH 7.4) and sonicated for two cycles (5 min, 50% amplitude, 0.3 s on, 0.7 s off). After sonication, the solution was centrifuged, and the supernatant was purified using TALON Superflow beads. Proteins were eluted with 300 mM imidazole, and imidazole was removed using a 3 kDa centrifugal filter (Millipore). The protein concentration was measured at 280 nm, and the protein was stored at −80 °C for long-term use.

DCLK3 can also be purified using immobilized metal affinity chromatography (IMAC) on a GE AKTA Start system with a 5 ml Cytiva HisTrap FF column. The binding buffer consisted of 50 mM HEPES, 150 mM NaCl, and 15% glycerol at pH 7.4. DCLK3 was extracted using an elution buffer containing 50 mM HEPES, 500 mM imidazole, 150 mM NaCl, and 15% glycerol at pH 7.4. The imidazole concentration was gradually increased over 10 column volumes, and 1 ml fractions were collected. The kinase-dead DCLK3 (D499A) construct was created using the Q5 site-directed mutagenesis kit (NEB) and purified following the same protocol.

### 3R Tau expression and purification

The 3R Tau construct was a gift from the Kanthasamy lab at the University of Georgia. It contains a cysteine to serine mutation at residue 322 and was inserted between NdeI and XhoI in the pET-28a expression vector with a hexahistidine tag at the N-terminus (GenScript). The HIS-tagged protein was purified using methods described previously (38), with an additional size-exclusion chromatography step. After elution with imidazole, the fractions were checked on SDS-PAGE, and those containing 3R Tau were further purified using a HiLoad Superdex SEC column (Cytiva) with elution in 50 mM HEPES, 150 mM NaCl, 15% glycerol at pH 7.4. Since 3R Tau lacks tryptophan residues, each fraction was checked by SDS-PAGE for protein identification. Fractions containing 3R Tau were pooled, protein concentration was measured using the Bradford assay, and the protein was stored at −80 °C for long-term use.

### DCLK expression data pre-processing and visualization

The data for Figure 1, *C* and *D* were downloaded from a publicly available CELLxGENE database (https://cellxgene.cziscience.com/), which is a collection of various tissues from 1764 single-cell RNA-sequencing datasets (34). The gene expression dataset was filtered for human brain tissue for DCLK1, DCLK2, and DCLK3. The comma-separated value (csv) file format was downloaded and visualized using scripts written in the R programming language. Figure 1, *C* and *D* were generated using ggplot2 (49) and dplyr in R (50). Figure 1*C* shows the expression of 30 major neuronal cell types, but the extended dot plot showing the expression of all 176 brain cell types is provided in Fig. S17. The expression of DCLK3 across multiple cancer types compared with normal tissue is provided in Fig. S16, (51). The input file includes the expression value for each gene per brain cell type along with the number cells expressing that gene. The percentage of cells expressing each DCLK was calculated using the formula below:

$$Expressed\;cells\;\%\;for\;gene\;X\;in\;cell\;type\;Y=\frac{Cells\;expressing\;gene\;X}{Total\;cells\;in\;cell\;type\;Y}\times 100\%$$

The data used for the protein expression analysis (Fig. S11) is downloaded from the publicly available HPA (https://www.proteinatlas.org/) portal (52). The input dataset is a CSV file downloaded from HPA based on The Human Protein Atlas version 4.1 and Ensembl version 54.36 (48). The raw data file is expression profiles for protein based on immunohistochemistry using tissue microarrays. The file includes antibody identifiers (hpa_id), Ensembl gene identifier (ensembl_gene_id), tissue name, intensity (negative, weak, moderate, or strong), fraction and the summary expression value. The dataset was then subsetted for only DCLK1, DCLK2 and DCLK3 gene and protein entries using ensemble ids ‘ENSG00000133083,’ ‘ENSG00000141564,’ and ‘ENSG00000128342,’ respectively. The protein names on the x axis and label tissues on the y axis were used to generate this heatmap.

### Differential scanning fluorimetry

Wild-type and KD (D499A) DCLK3 at a final concentration of 0.35 mg/ml were mixed with 1:250 SYPRO Orange (Sigma) dye in a buffer containing 50 mM HEPES (pH 7.4), 150 mM NaCl, and 10% glycerol. ATP and MgCl_2_ were added at final concentrations of 2 mM and 4 mM, respectively. The samples were gradually heated from 20 °C to 95 °C over the course of 2 h on a StepOne Plus Real-Time PCR system (Thermo Fischer Scientific), and the data were analyzed in Prism 10 (GraphPad). The experiment was repeated three times under the same conditions.

### ADP-Glo assay

ADP-Glo kinase assays were performed according to manufacturer's protocol (Promega). WT- and KD-DCLK3 (0.2 mg/ml) were added to the kinase reaction in the presence and absence of 3R Tau (0.4 mg/ml). Luminescence was detected 1 h after adding Kinase Detection Reagent on a Biotek Synergy HT plate reader. Data was analyzed in Prism 10 (GraphPad). The experiment was repeated three times under the same conditions.

### Prediction of DCLK3 substrates

DCLK3 substrates were predicted using Phosformer-ST, a protein language model fine-tuned on experimentally validated peptide arrays for nearly 300 serine/threonine kinases within the human genome (1, 23). Since DCLK3 is an understudied kinase not included in the training set, the model’s zero-shot prediction capability was leveraged. The kinase domain of DCLK3 was used as input, along with a curated set of phospho-serine and phospho-threonine peptides derived from the human proteome (23, 37). Peptides scoring above 0.98 on the model's confidence metric were classified as high-confidence DCLK3 substrates and subsequently mapped to their respective full-length proteins. Gene Ontology (GO) analysis was performed on these proteins to identify functional annotations, particularly focusing on those associated with “microtubule binding”. Proteins meeting these criteria, including Tau, are presented in Fig. S7.

Phosphorylation sites within Tau (2N4R isoform) were further analyzed by running all 15-mer peptides containing serine or threonine residues in the central position through Phosformer-ST. Input included the kinase domains of human DCLK1, DCLK2, and DCLK3, and all 15-mer peptides from 2N4R Tau. Peptides were ranked by predicted phosphorylation scores for DCLK3, and peptides with corresponding scores are shown in Table S2.

### *In vitro* kinase assay

For the *in vitro* kinase assay, 24 μl of 0.5 mg/ml WT- or KD-DCLK3 was placed in a 1.5 ml centrifuge tube, followed by 6 μl of 5× kinase buffer (200 mM HEPES, 200 mM NaCl, 10% Glycerol, 100 mM MgCl2) containing 5 mM ATP. The reaction was incubated at 30 °C for the indicated times. To stop the reaction, 10 μl of 4× Laemmli buffer was added, and the mixture was heated at 95 °C for 5 min. Proteins were separated on 10% SDS-PAGE, transferred to a PVDF membrane using the Bio-Rad Trans-Blot Turbo system, and blocked with 3% BSA for 1 h. Immunoblotting was performed with anti-HIS antibody (Cell Signaling Technology) and HRP-conjugated secondary antibody (Jackson Laboratory). Protein bands were visualized using a chemiluminescent substrate and the LICOR Odyssey M instrument.

### Protein modeling and simulation

The DCLK1 crystal structure was obtained from the RCSB Protein Data Bank (RCSB PDB) (PDBID: 6KYQ) (53). All other structural conformations of kinases, such as DCLK2, DCLK3, and pDCLK3, were generated using AF3. Amino acid sequences of full-length proteins were obtained from UniProt (https://www.uniprot.org/) (54). Sequence inputs are depicted in Table S3.

To generate the conformational ensemble of kinases, we used a local implementation of AF3. The required model parameters of AF3 were requested from Google DeepMind. The sequences of protein construct were provided to the AF3 model under two conditions: first, DCLK3, without PTMs (representing the unphosphorylated form), and second, pDCLK3, with PTMs (phosphorylated to the five residues). 50 seeds were used for each condition, where AF3 generates five varying ranked models per seed, resulting in 250 predicted structures. The conformational distribution was clustered based on the RMSD of all pairwise structures. In both cases, three primary clusters of distinct structures were identified (Fig. S6). The most probable predicted conformation from AF3, DCLK3 (39.60%) and pDCLK3 (61.20%) were used as the initial configuration to set up and run MD simulations. Simulation details are shown in Table S4.

### Simulation parameters

GROMACS 2023.3 package was used for the simulation setup and data production (55). A cubic simulation box was constructed, ensuring a minimum padding of 1.5 nm from the protein structures. The system was solvated using the TIP3P water model, and NaCl was added to maintain a physiological ionic concentration of 150 mM and achieve system neutralization (56). Energy minimization was done for all the systems with the steepest descent algorithm for a maximum of 600,000 steps. The systems were equilibrated under isothermal-isochoric conditions (NVT ensemble, T = 310 K) and followed by isothermal-isobaric conditions (NPT ensemble, T = 310 K), each for 100 ps. The production of all the systems was run at 2 fs time step for three sets, with a total simulation time of 500 ns. Particle-mesh Ewald (PME) summation method was used for the calculation of long-range electrostatic interactions (57), and the HARMM36 force field was used for all the simulations (58). The resulting generated data were visualized and analyzed using MDAnalysis (59), VMD (60), and PyMOL (61).

### Proteolysis and LC-MS analysis

Each MS protein sample was separated by SDS-PAGE and stained with Coomassie Blue. Bands corresponding to the molecular weights of the respective constructs (3R Tau, WT-DCLK3, KD-DCLK3, DCLK3 + Ubiquitin) were excised, as shown in Figs. S5 and S14. The excised bands were reduced by incubating with 5 mM dithiothreitol (Sigma) at 56 °C, alkylated with 13.75 mM iodoacetamide (Sigma) at room temperature in the dark, and then digested overnight at 37 °C using a trypsin/LysC mix (Promega). After digestion, the peptides were extracted and dried.

The peptides were separated on an Acclaim PepMap 100 C18 column (75 μm × 15 cm) and eluted into the nano-electrospray ion source of an Orbitrap Eclipse Tribrid mass spectrometer (Thermo Scientific) at a flow rate of 200 nl/min. The spray voltage was set to 2.2 kV and the temperature of the heated capillary was set to 275 °C. Full MS scans were acquired from m/z 300 to 2000 at 60k resolution, and MS/MS scans following either collision-induced dissociation (CID) with multistage activation, or higher-energy collisional dissociation (HCD) with stepped collision energy (15%, 25%, 35%), were collected in the iontrap or orbitrap at 15k resolution, respectively. The spectra were analyzed using SEQUEST (Proteome Discoverer 2.5, Thermo Fisher Scientific) with mass tolerance set as 20 ppm for precursors, and 0.5 Da (iontrap) or 20 ppm (orbitrap) for fragments. The search output was filtered to reach a 1% false discovery rate at the protein level and 10% at the peptide level. The quantitation was performed based on spectral counts.

### Transfection, immunoprecipitation, and Western blotting

Mammalian plasmids, FLAG-vector, GFP-vector and FLAG-DCLK3 were purchased from Genecopoeia, while GFP-Tau, and HA-Ub were obtained from Addgene. HEK293T cells (ATCC) were transfected with plasmids using the PEI transfection method. Briefly, cells were grown to 50% confluency on 6 cm tissue culture plates in antibiotic-free DMEM media. 10 μg of plasmid was incubated with 20 μl of PEI in 500 μl OPTI-MEM (Gibco) for 15 min, then added to the plate containing 2 ml of media. For DCLK3 and GFP-Tau interaction/phosphorylation, cells were co-transfected with the indicated plasmids. After 48 h, the cells were lysed in RIPA buffer. Cell lysates were subjected to co-immunoprecipitation using anti-FLAG M2 magnetic or agarose beads (both worked), followed by washing the bound proteins three times in RIPA buffer and eluting them in SDS-PAGE sample buffer. The proteins were separated on a 10% SDS-PAGE gel, transferred to a PVDF membrane, and immunoblotted with the specified antibodies (all antibodies were purchased from Cell Signaling Technologies). The proteins were visualized using a LICOR Odyssey system, and signal analysis software was used for quantification.

### Proteasome inhibitor (MG132) treatment and cycloheximide chase assay

HEK293T cells were transfected with FLAG-vector or FLAG-DCLK3 on 6 cm plates as described above. 48 h after transfection, cells were treated with 0 to 500 nM MG132 (Calbiochem) for 16 h, with DMSO as a control for 0 nM treatment. Cells were lysed in 1 ml RIPA buffer, and protein concentration was measured by UV280, adjusting to 1 mg/ml for all samples. Samples were separated on a 10% SDS-PAGE gel, transferred to a PVDF membrane, and immunoblotted with anti-FLAG antibody. GAPDH was used as a loading control and detected with an anti-GAPDH antibody. Both proteins were detected on the same blot by using a mixture of antibodies (Fig. S12).

For the cycloheximide chase assay, transfection, cell lysis, and protein determination were done as described earlier. Cells were treated with 10 μg/ml cycloheximide and harvested at the indicated time points. Protein levels were examined by immunoblotting with anti-FLAG and anti-GAPDH antibodies (Figs. S12 and S14).

## Data availability

The mass spectrometry data have been deposited to the ProteomeXchange Consortium *via* the MassIVE partner repository with the dataset identifier MSV000097817 (https://massive.ucsd.edu/ProteoSAFe/static/massive.jsp). All other data needed to support the finding in the paper are available within the manuscript and its Supplementary Information files.

## Supporting information

Supplementary information is available for this paper.

## Conflict of interest

The authors declare that they have no conflicts of interest with the contents of this article.
