# Supplementary material for: Deciphering the unique autoregulatory mechanisms and substrate specificity of the understudied DCLK3 kinase linked to neurodegenerative diseases
Source: J Biol Chem. 2025 Sep 1;301(10):110664. doi: 10.1016/j.jbc.2025.110664 (PMC12509982; doi:10.1016/j.jbc.2025.110664)
Supplement: Supporting Figures [file mmc1.docx]

**Supplementary Figures for**

**Deciphering the unique autoregulatory mechanisms and substrate specificity of the understudied DCLK3 kinase linked to neurodegenerative diseases**

Jason D. Lu^1^, Peng Zhao^2^, Anup Prasad^1^, Neha Gupta^3^, Nathan Gravel^3^, Tej P. Shidhaye^1^, Lance Wells^1,2^, Samiksha Katiyar^3^* and Natarajan Kannan^1,3^*

^1^Department of Biochemistry and Molecular Biology, University of Georgia, Athens, GA 30602

^2^Complex Carbohydrate Research Center, University of Georgia, Athens, GA 30602

^3^Institute of Bioinformatics, University of Georgia, Athens, GA 30602

*Corresponding authors: Samiksha Katiyar ([samiksha@uga.edu](mailto:samiksha@uga.edu)) and Natarajan Kannan ([nkannan@uga.edu](mailto:nkannan@uga.edu))

**This file includes:**

Figs. S1 to S17


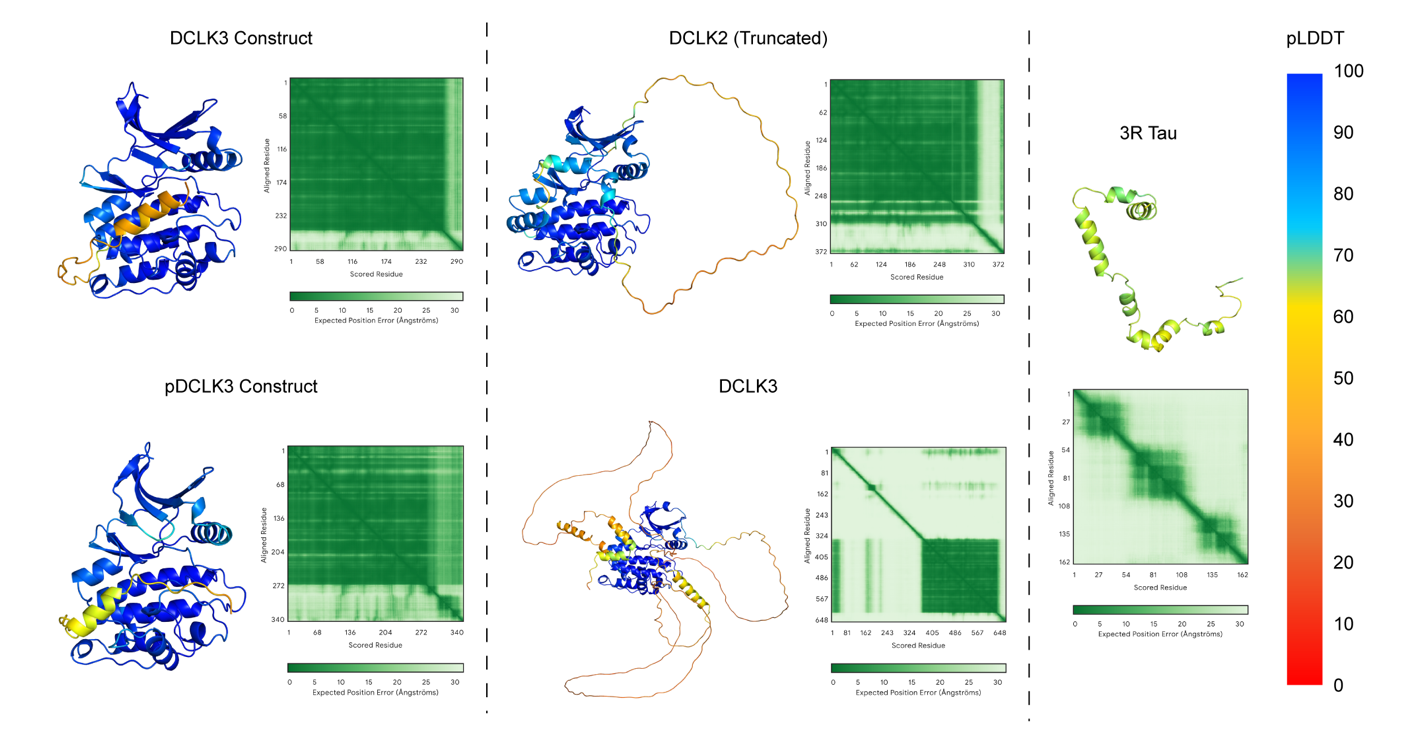

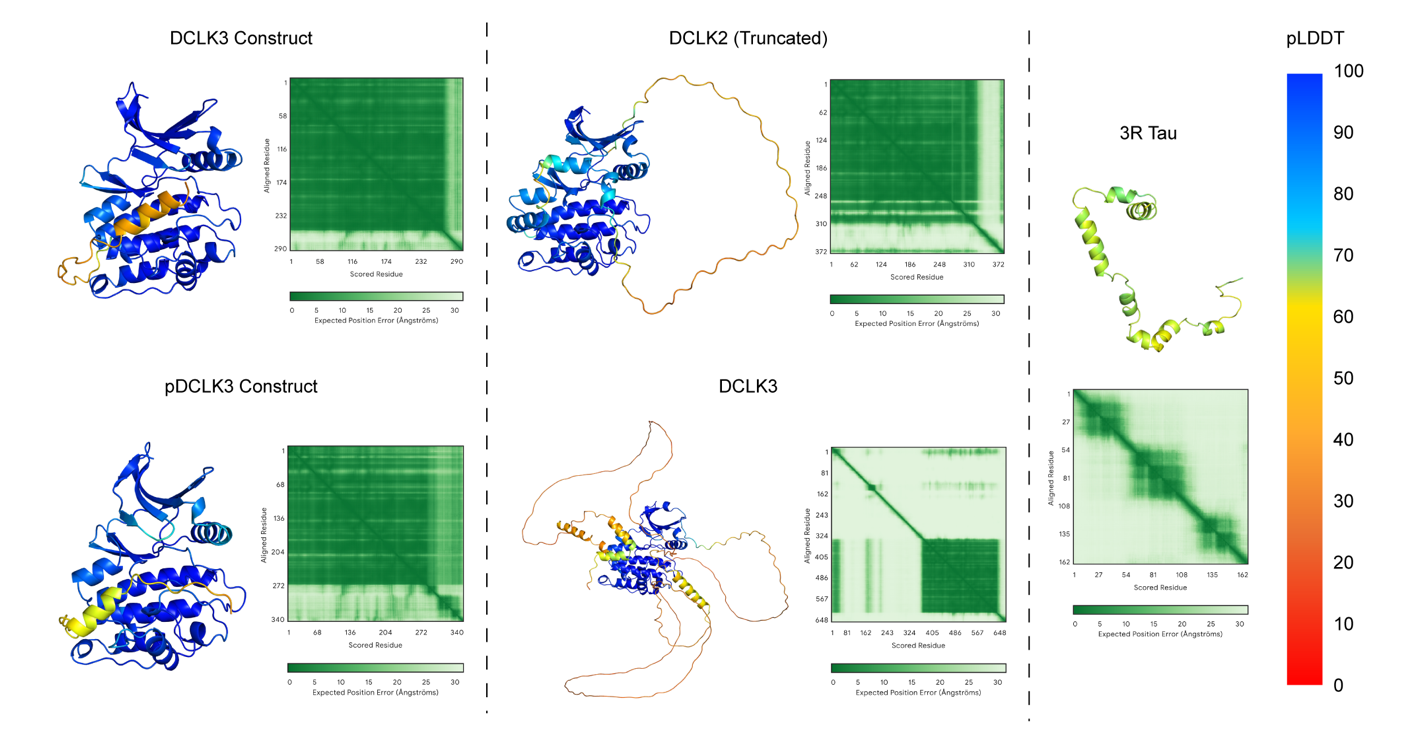


**Fig. S1.** AlphaFold 3 structural prediction pLDDT and PAE scores. Predicated structural models for indicated proteins: DCLK3 experimental construct (DCLK Construct), autophosphorylated DCLK3 experimental construct (pDCLK3 Construct), DCLK2 kinase domain and tail (DCLK2 (Truncated)), and full-length DCLK3 (DCLK3). Structures are colored according to their predicted local distance difference test (pLDDT) scores. Predicted Aligned Error (PAE) heatmaps are shown to the right of each structure, indicating expected positional error in Å.


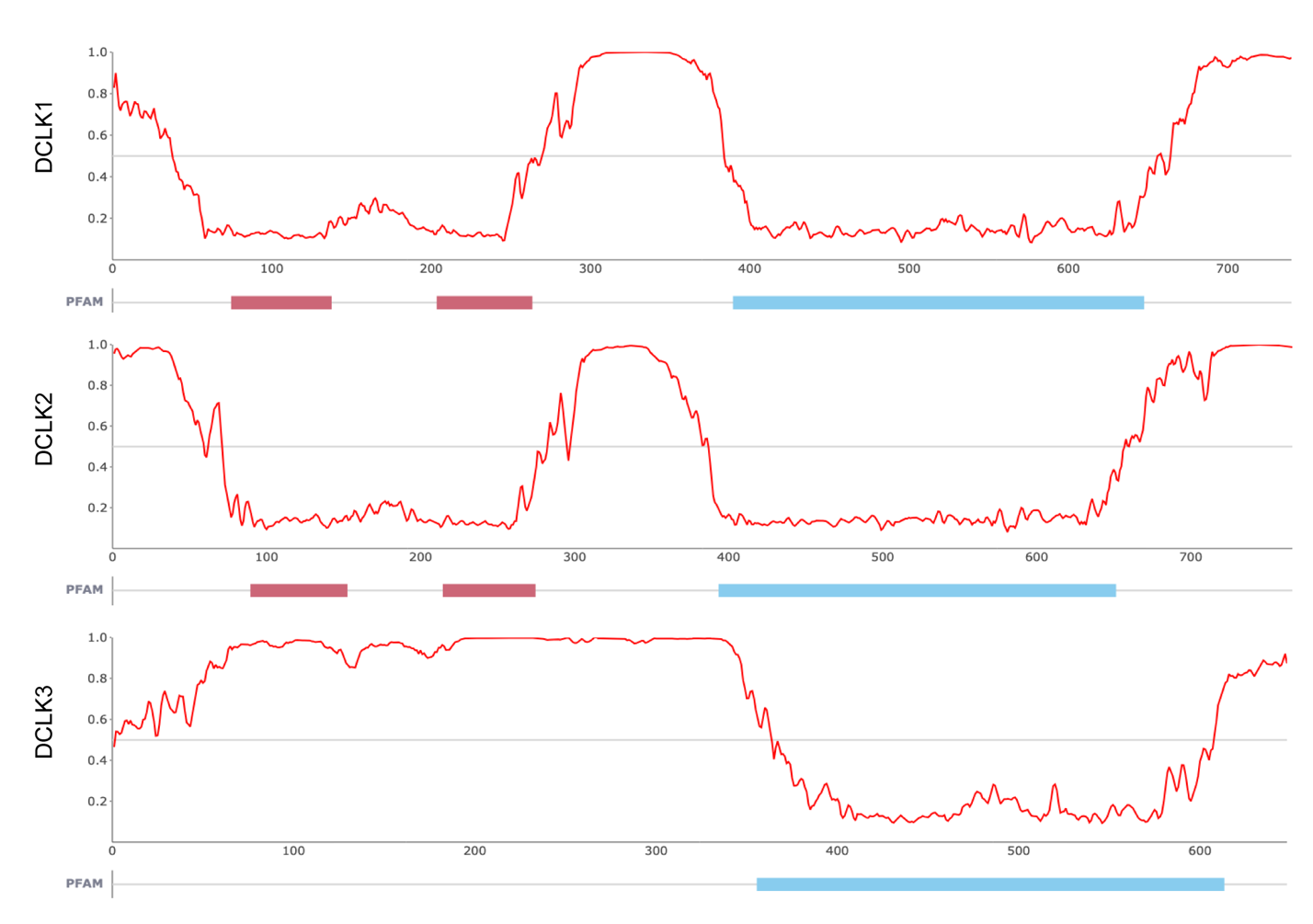
**Fig. S2.** Intrinsic disorder probability profiles for full-length DCLK1, DCLK2, and DCLK3 using AIUPred web tool (https://aiupred.elte.hu/). PFAM-annotated domains are shown below each profile.

**
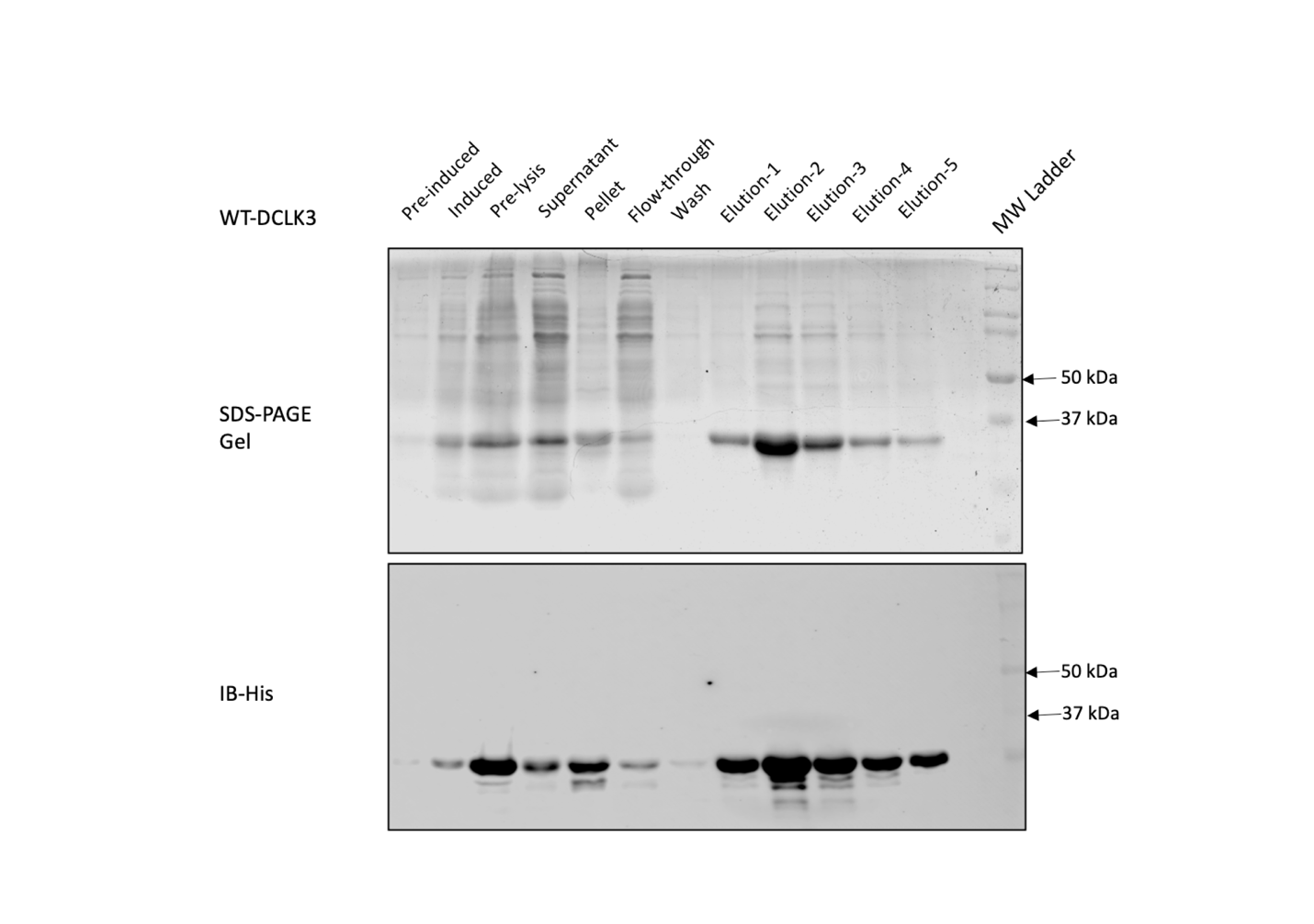
Fig. S3.** Full-length gel and blot corresponding to the SDS-PAGE and Western blot of DCLK3 Construct purification shown in Figure 2A.


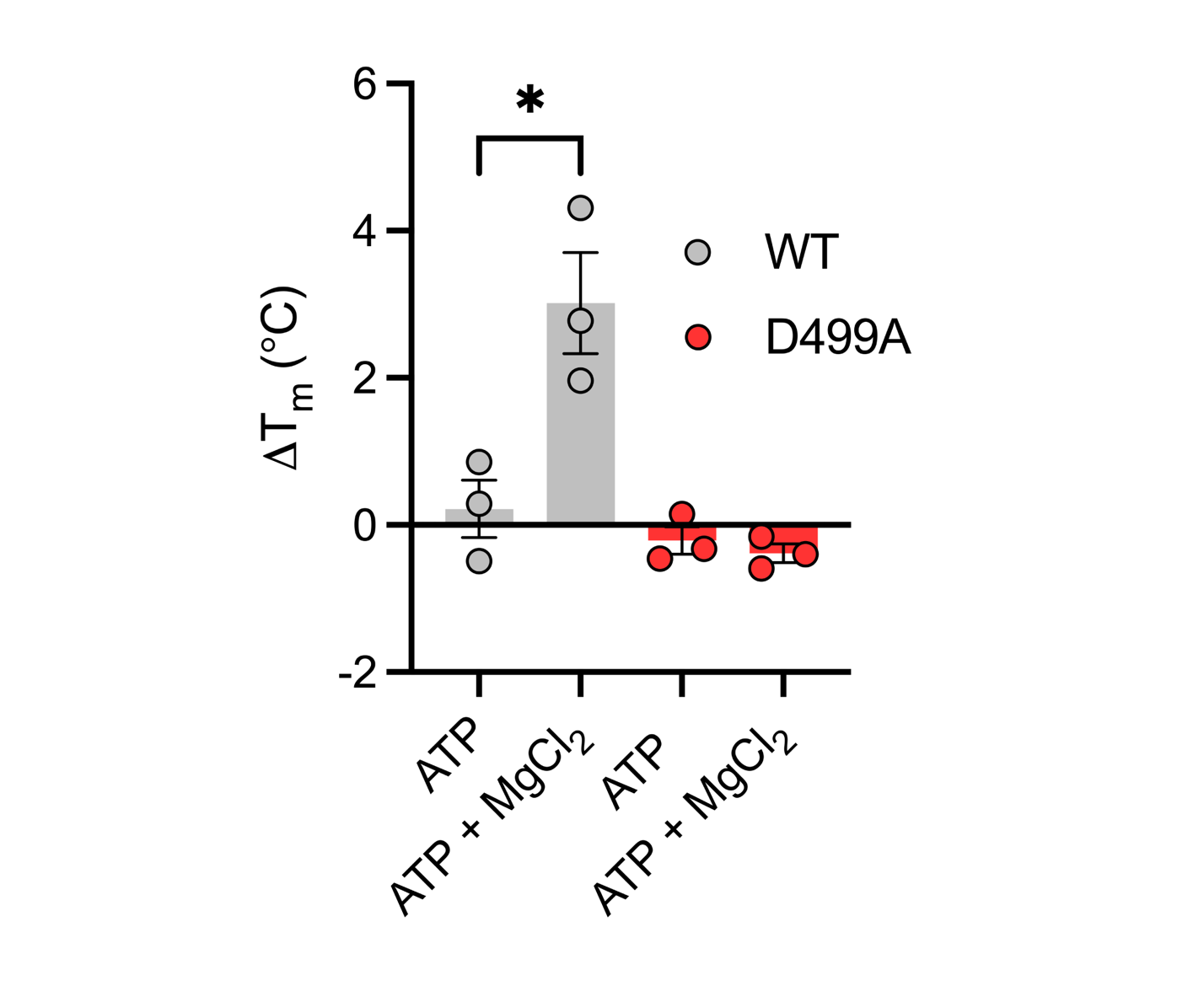
**Fig. S4.** Thermal shift in melting temperature (ΔTm) of WT and KD (D499A) DCLK3 in the presence of ATP or ATP and MgCl_2_. Data is represented as mean ± SEM (n = 3). Asterisk denotes statistical significance (*p* < 0.05). Statistical analysis was performed using a two-tailed unpaired t-test (*p* = 0.0242).

**
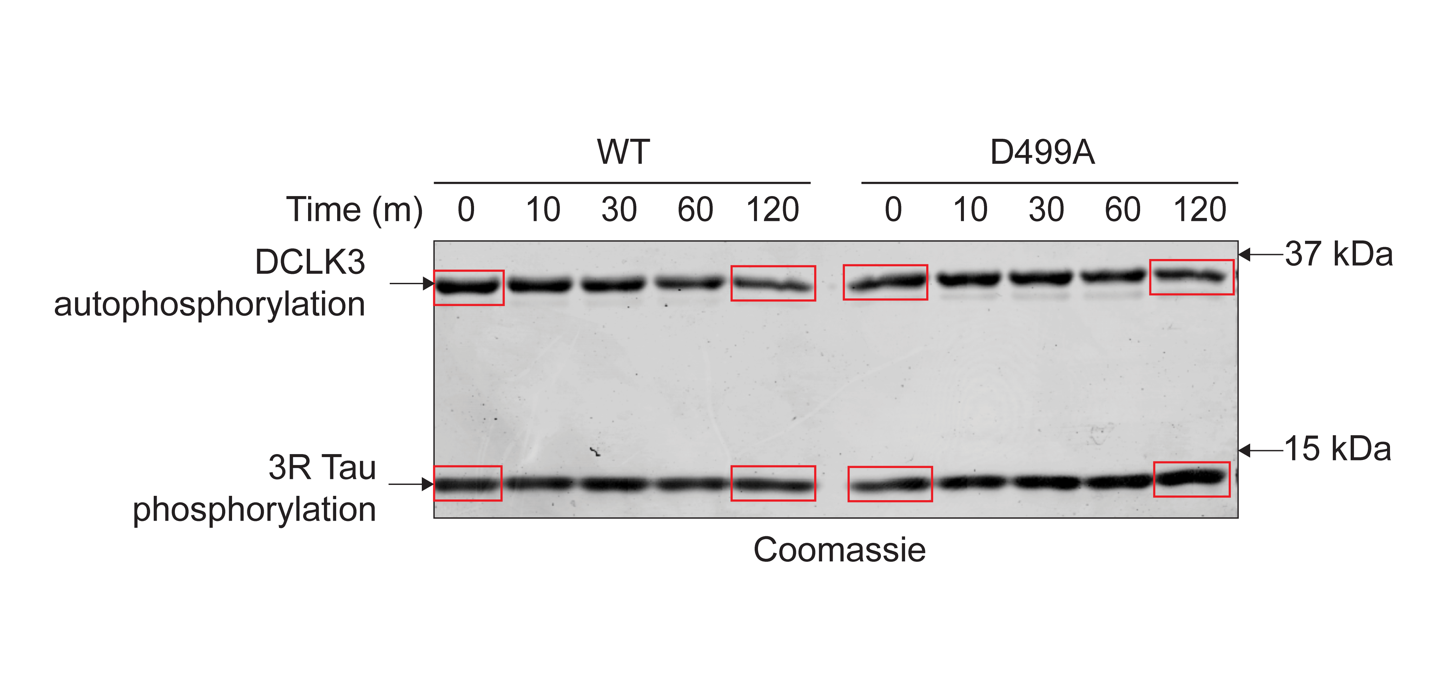
**

**Fig. S5.** SDS-PAGE Coomassie gel corresponding to the DCLK3 3R Tau kinase assay shown in Figure 4D. Samples excised for LC-MS/MS are indicated by the red boxes.

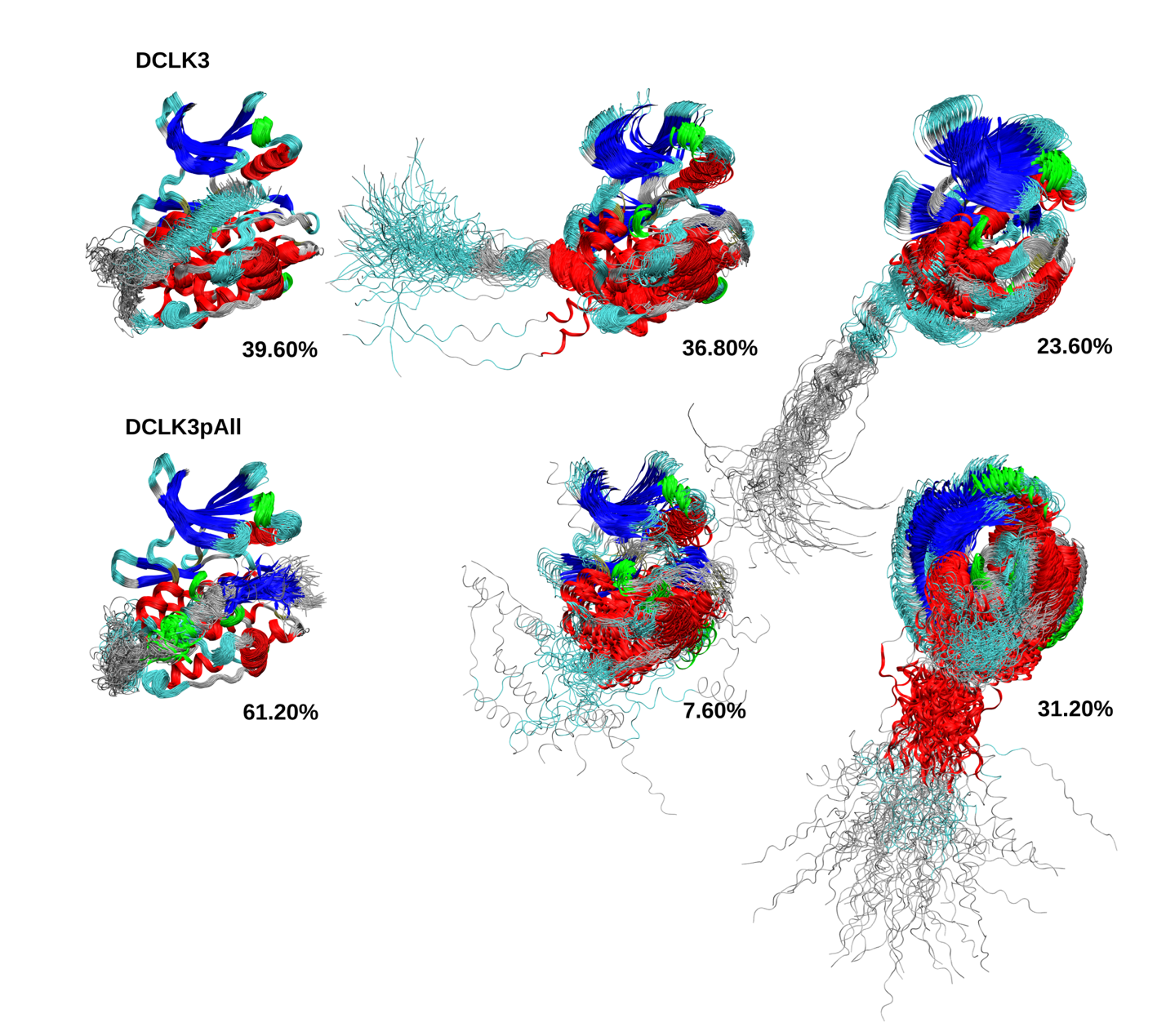
**Fig. S6.** AlphaFold 3 predicted structures of unphosphorylated (DCLK3) and phosphorylated (pDCLK3) kinases. AlphaFold 3 was used to generate a structural ensemble of a total of 250 conformers. These structures were clustered based on RMSD values of all the conformer populations. DCLK3 and pDCLK3 showed the most probable structure containing 39.60% and 61.20% conformers of the total generated populations, respectively. Interestingly, the C-tail is attached to the catalytic domain in the most probable structures of both cases. The higher probability of the C-tail-bound form in pDCLK3 also suggests the role of phosphates in structural stability.

**
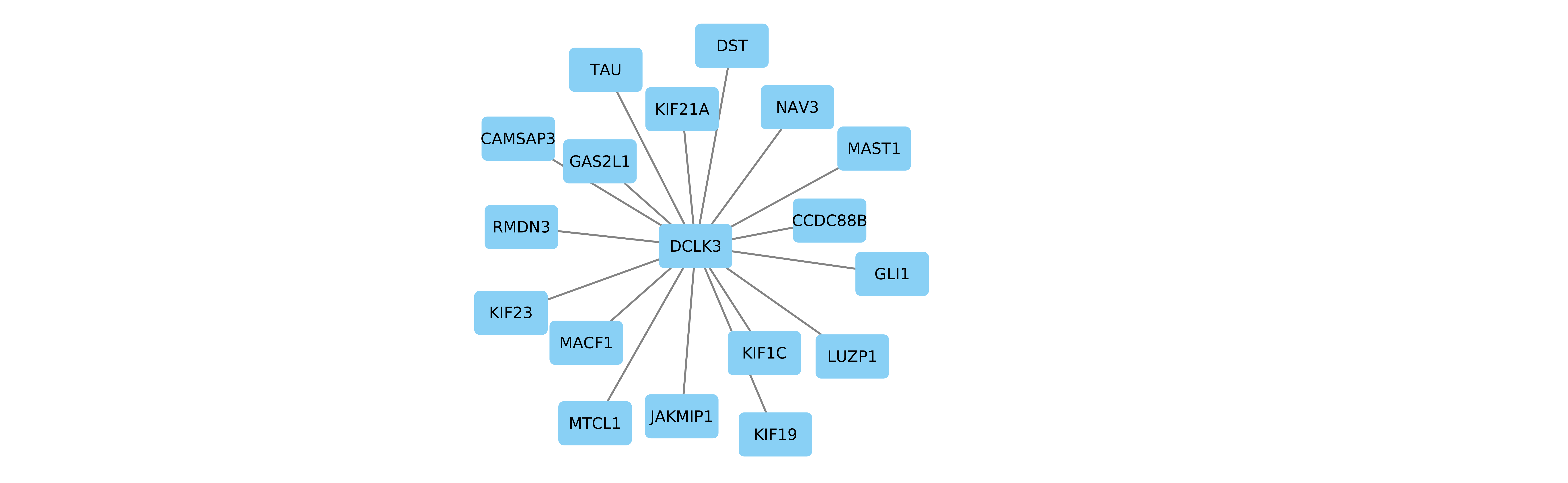
 (D) Fig. S7.** Phosfomer-ST predicted Cytoscape interaction network for DCLK3 (score >0. 98). The potential substrates are gathered from predicting on all existing substrates retrieved from the atlas of the human serine/threonine kinome dataset (23). The DCLK3 substrate candidates were then additionally filtered based on Gene Ontology analysis. Sustrates associated with the GO term “microtubule binding” were used. The resulting kinase-substrate network was plotted using Cytoscape. Predicted substrates are listed in Table S1.

**
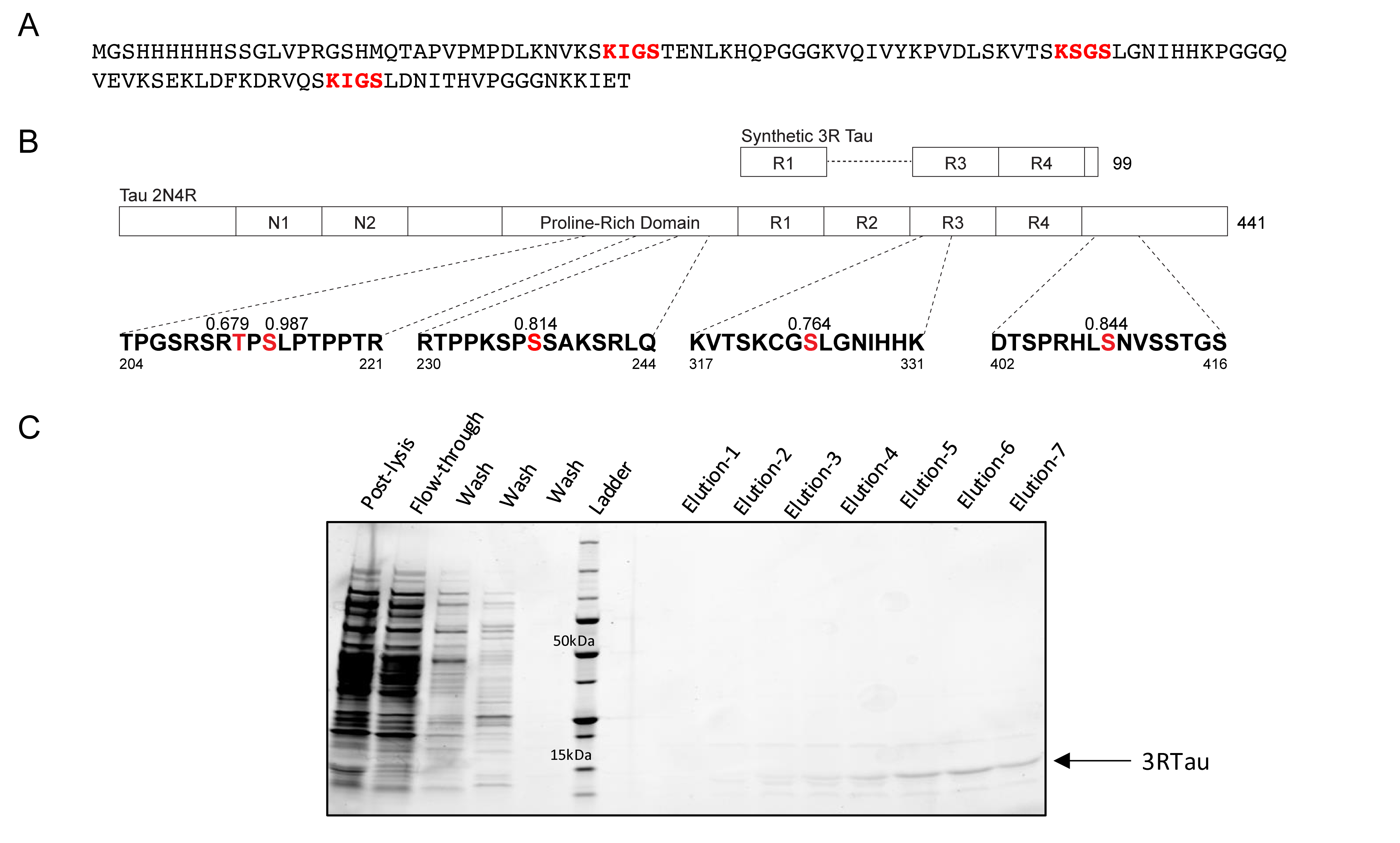
Fig. S8. (A)** Sequence of synthetic 3R Tau construct with KXGS motifs highlighted in red. **(B)** Domain architecture of synthetic 3R Tau alongside 2N4R Tau. **(C)** SDS-PAGE Coomassie gel of recombinant HIS-tagged 3R Tau purification. MW ladder positions are indicated.

**
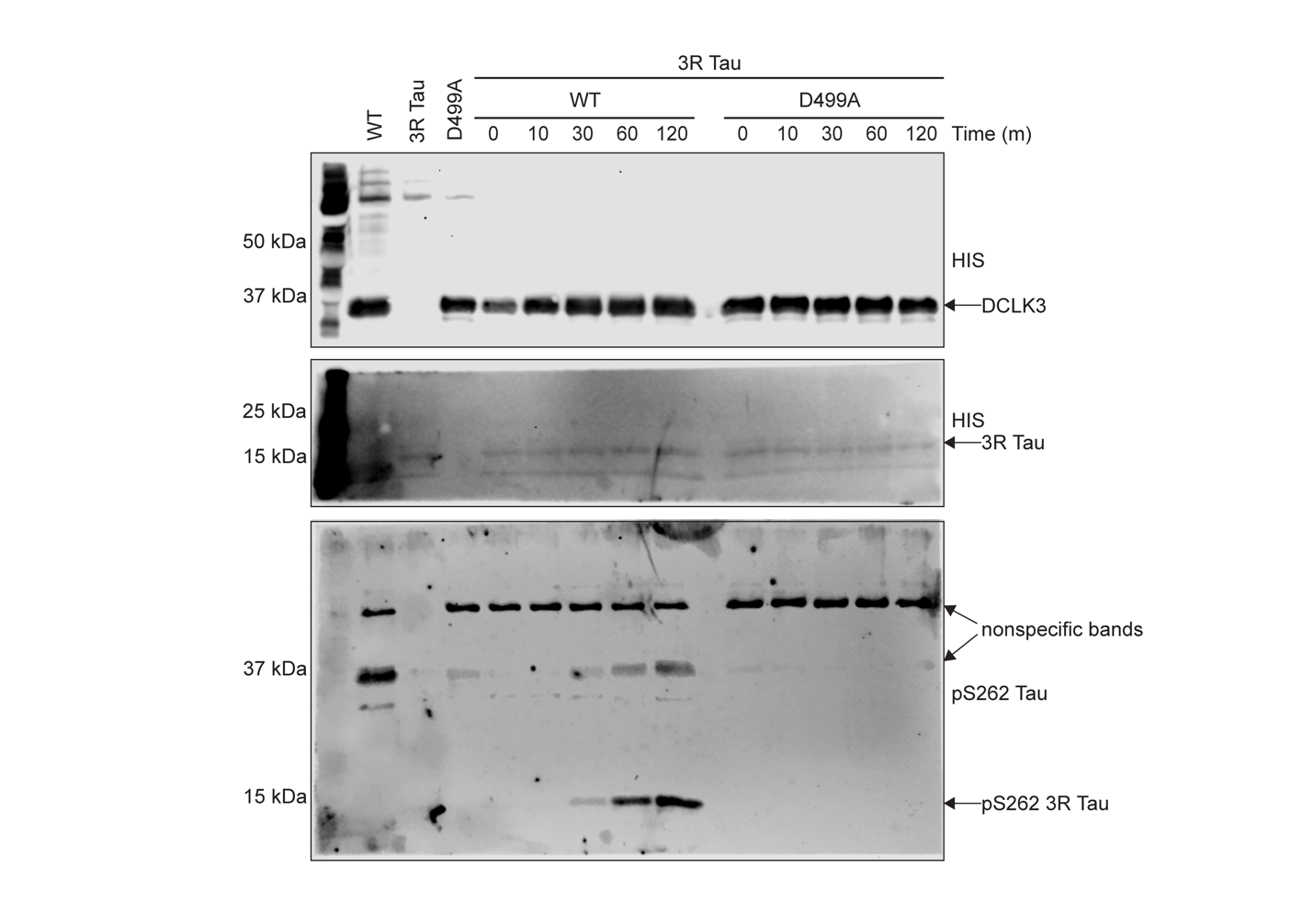
Fig. S9.** Full-length blots corresponding to the DCLK3 3R Tau kinase assay shown in Figure 4D.

**
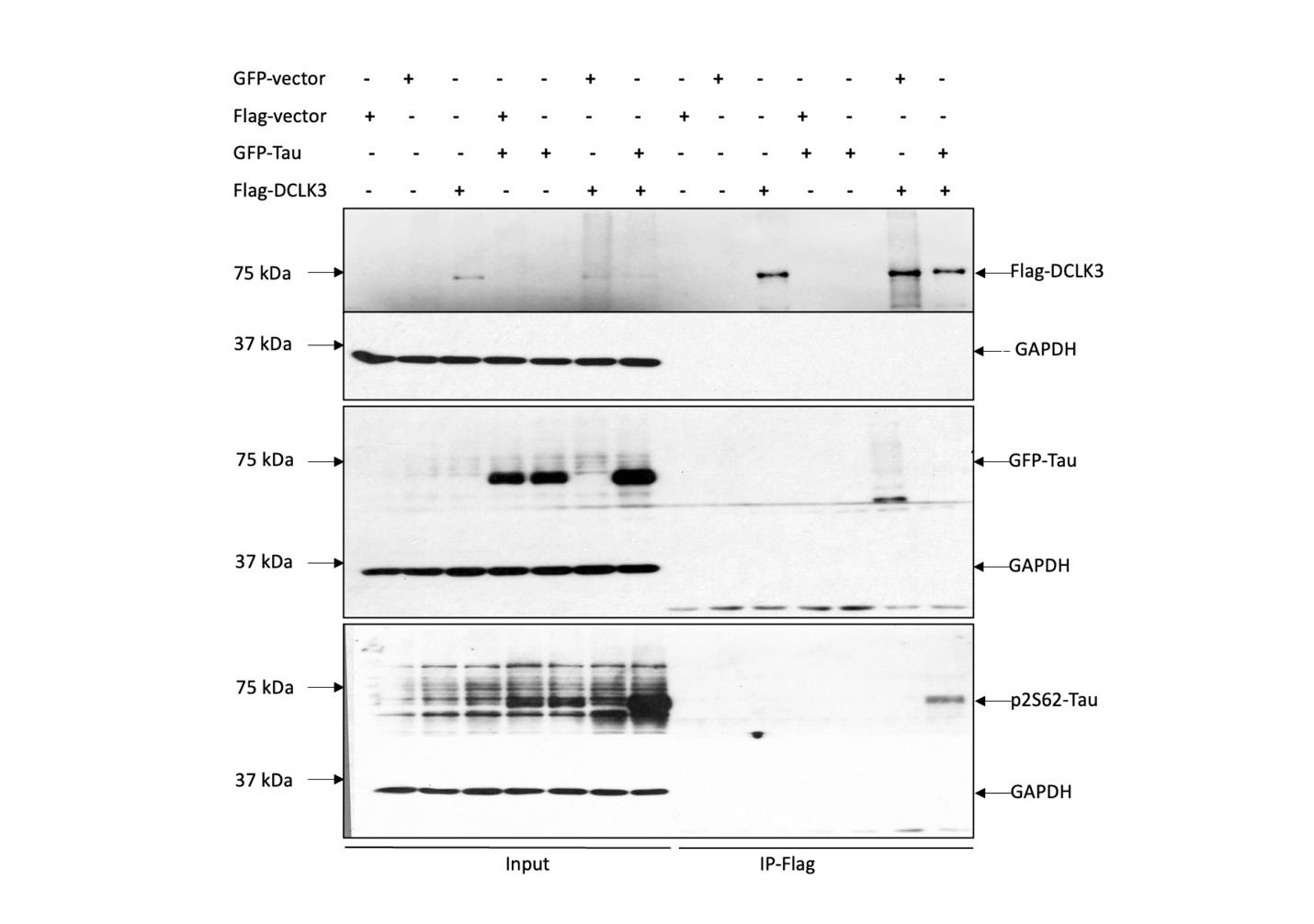
Fig. S10.** Full-length blots corresponding to the GFP-Tau FLAG-DCLK3 coimmunoprecipitation shown in Figure 4F.


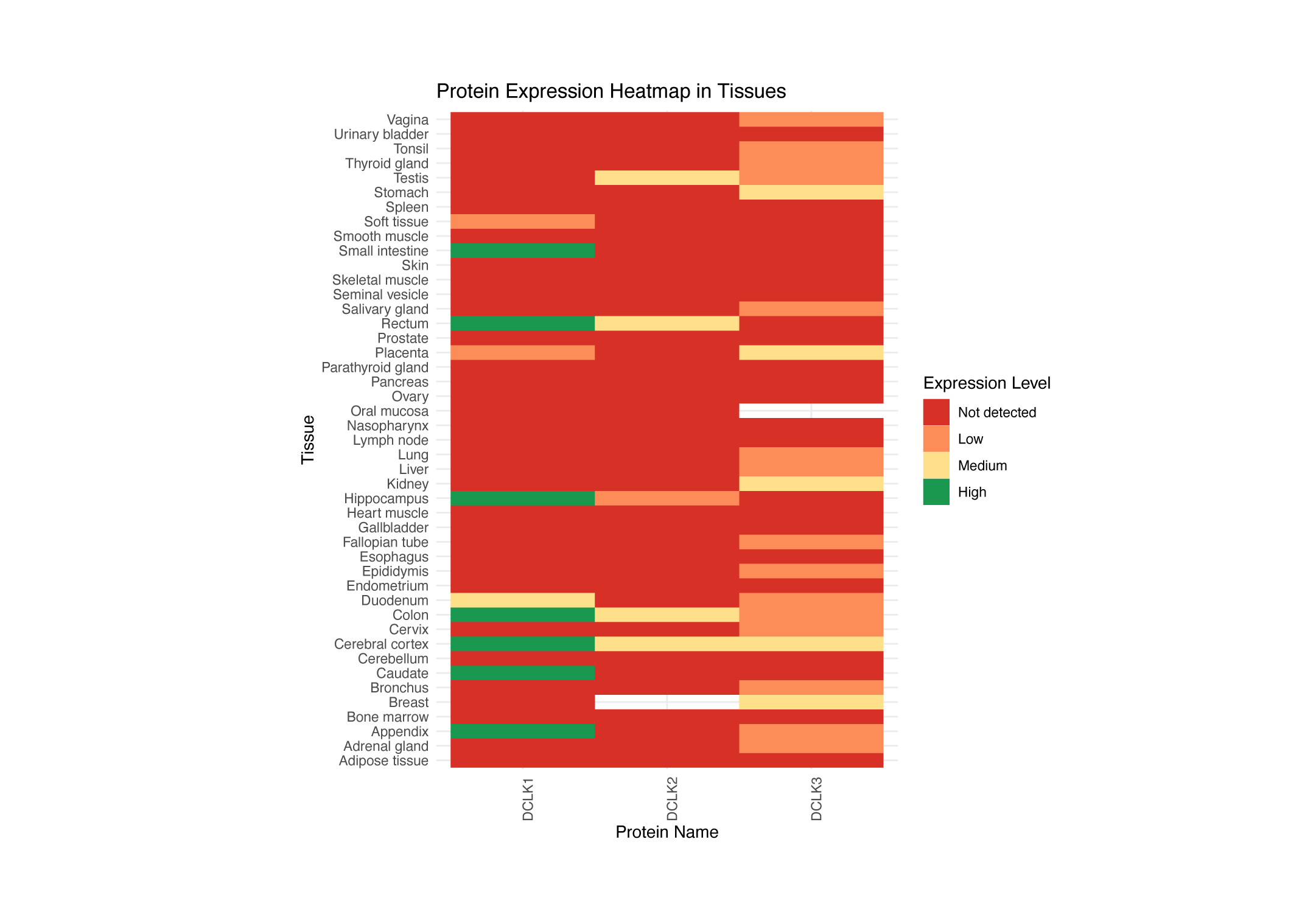
**Fig. S11.** Protein expression profile of DCLK3 paralogs across human tissues. Heatmap generated from data deposited on Human Protein Atlas showing relative expression levels of DCLK1, DCLK2, and DCLK3. Expression intensity is shown in green (high), yellow (medium), orange (low), red (not detected), or not shown (no data).

**
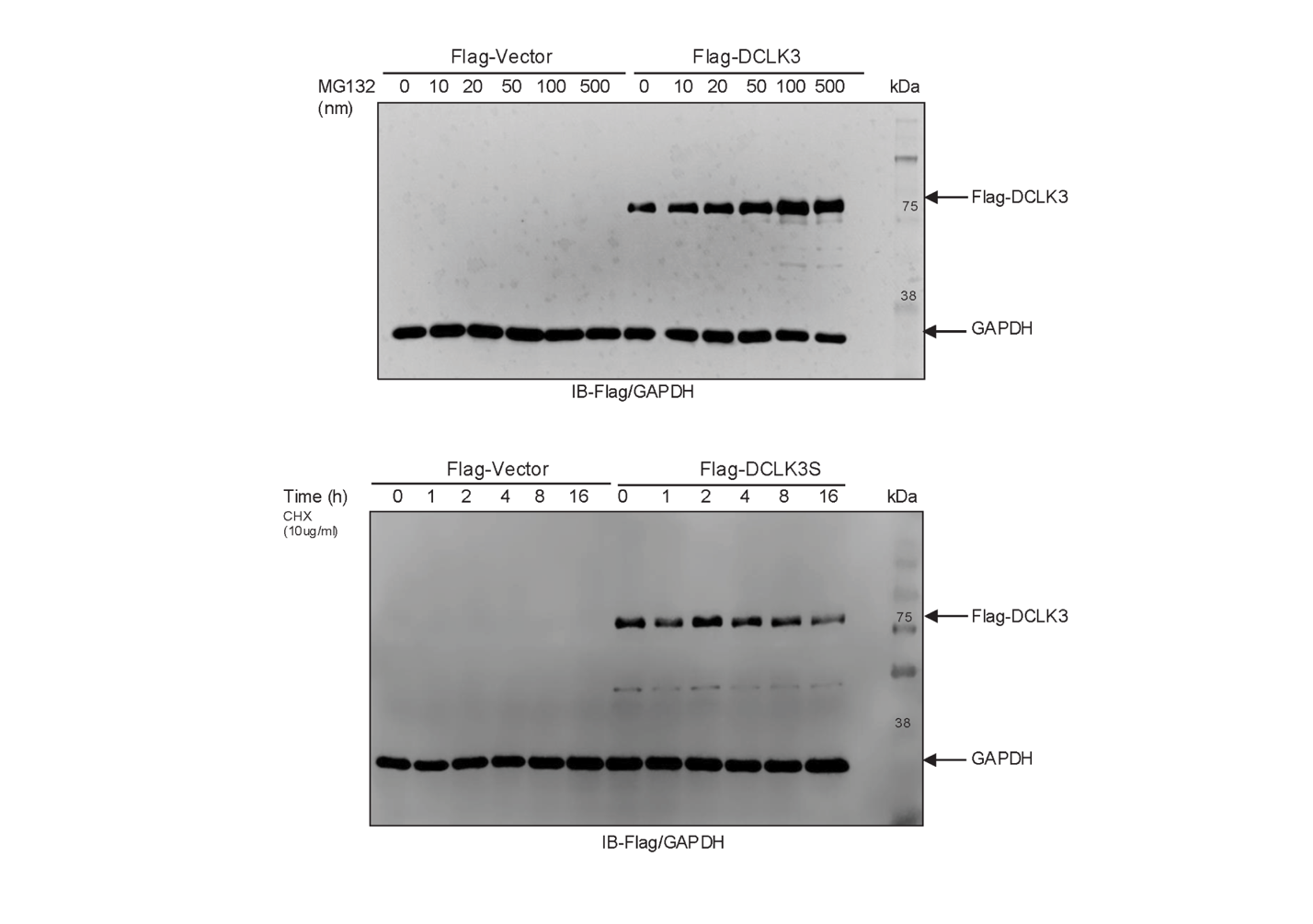
Fig. S12.** Full-length blots corresponding to the MG132 assay (top), shown in Figure 5A, and the CHX chase assay (bottom), shown in Figure 5C. Uncropped immunoblots detect no FLAG-tagged proteins in samples transfected with FLAG-Vector.

**
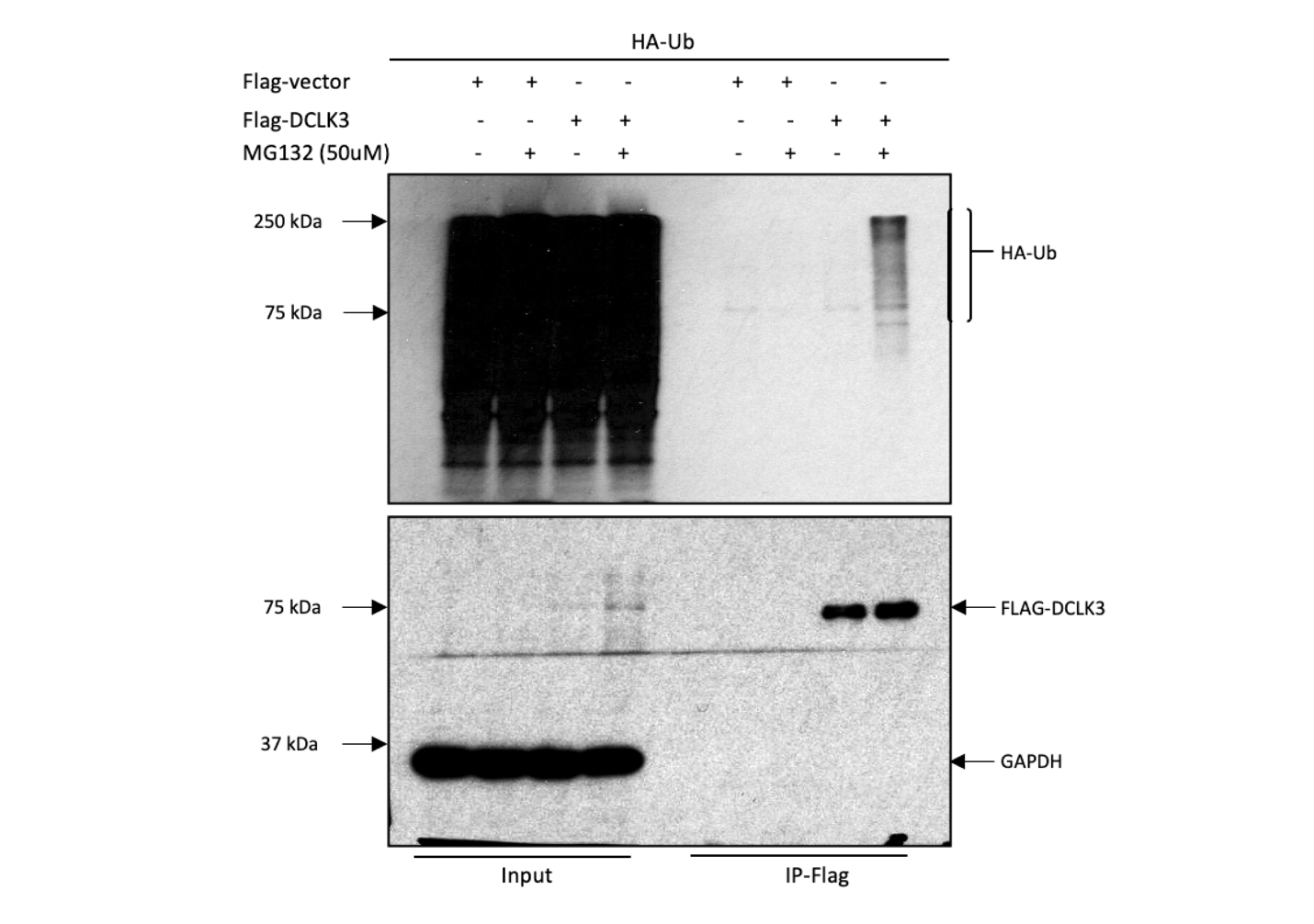
Fig. S13.** Full-length blots corresponding to the HA-Ubiquitin FLAG-DCLK3 coimmunoprecipitation shown in Figure 6A.

**
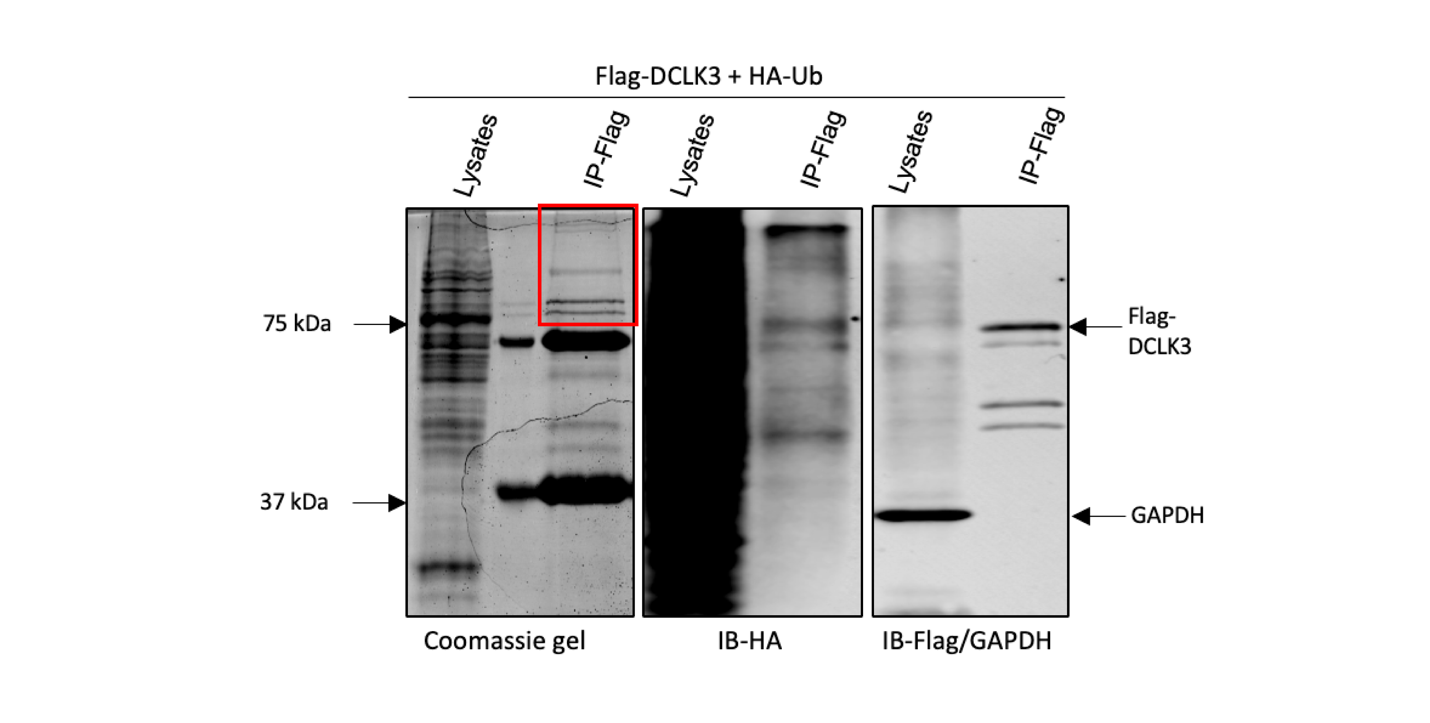
Fig. S14.** SDS-PAGE gel and Western blot of ubiquitinated FLAG-DCLK3 analyzed by LC-MS/MS. SDS-PAGE gel (left) and corresponding immunoblots probed with anti-HA (middle) and anti-FLAG (right) antibodies. Samples show whole-cell lysates (Lysates) and immunoprecipitates (IP-FLAG) of FLAG-DCLK3 co-transfected with HA-Ubiquitin. The area indicated by the red box was excised from the SDS-PAGE gel and analyzed by LC-MS/MS.


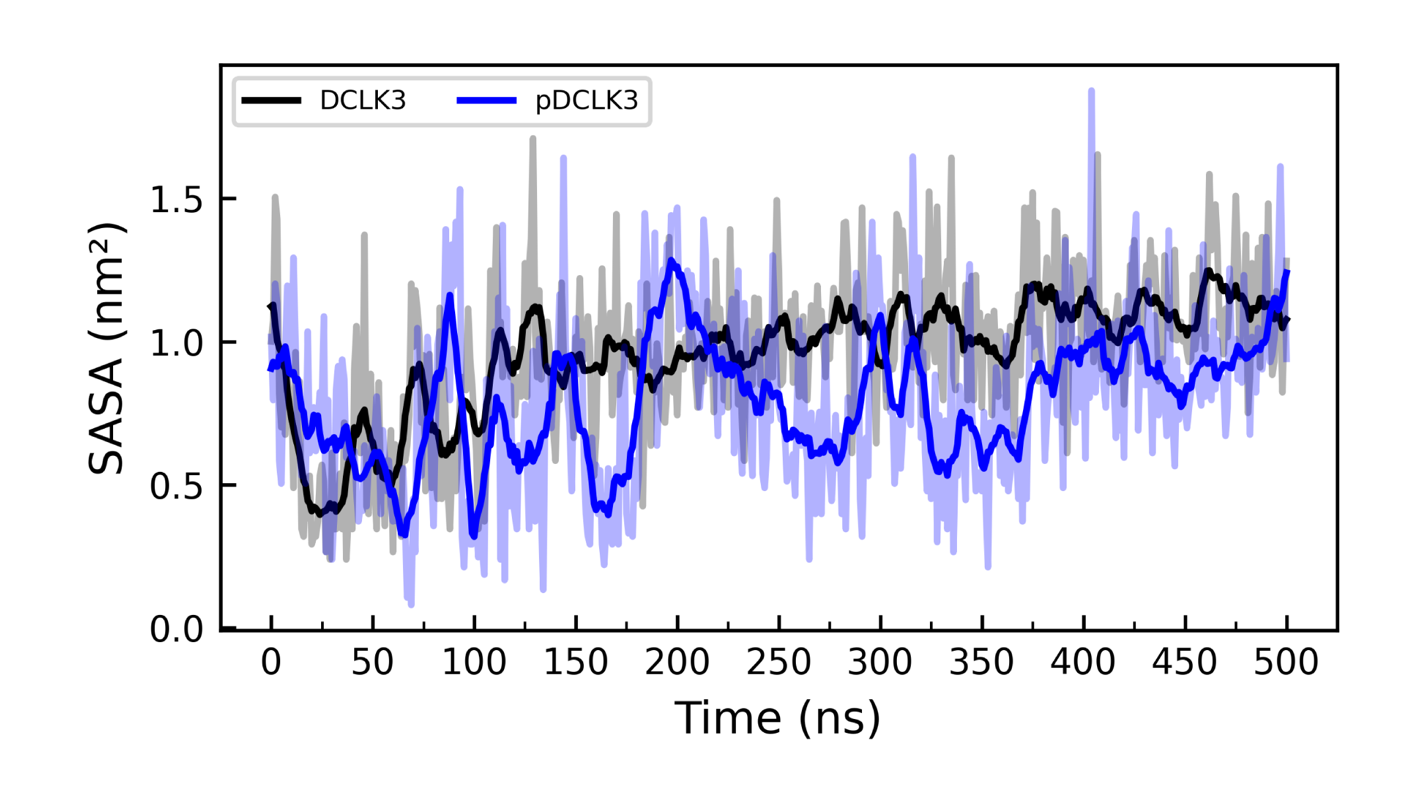
**Fig. S15.** Solvent accessible surface area (SASA) of K395 and K479 residues. The SASA analysis over the simulation trajectory revealed a significant difference between the unphosphorylated and phosphorylated forms of DCLK3. In the phosphorylated form (pDCLK3), the C-terminal tail, which interacts with the catalytic domain, shields residues K395 and K479, thereby reducing their solvent accessibility.

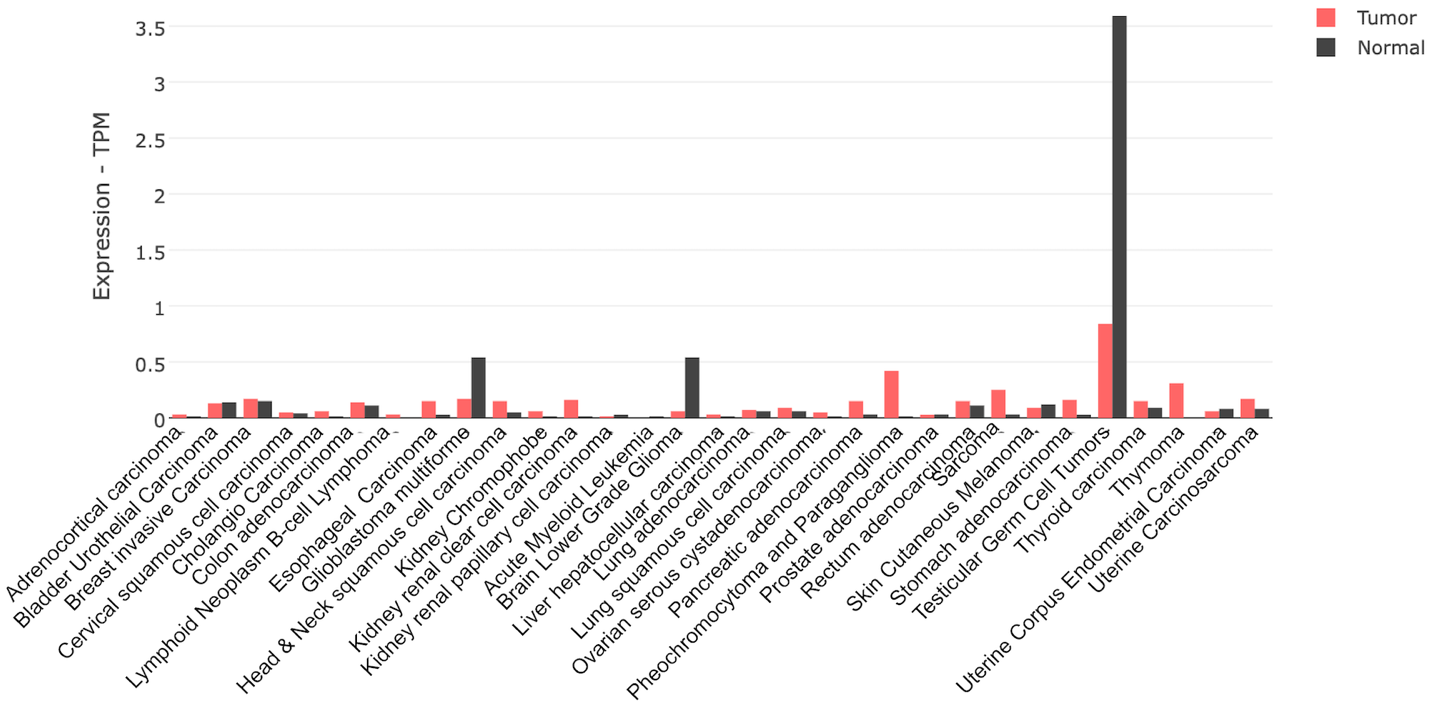


**Fig. S16.** DCLK3 expression in normal vs tumor tissue plotted on data from TCGA/GTEx using GEPIA2 portal.


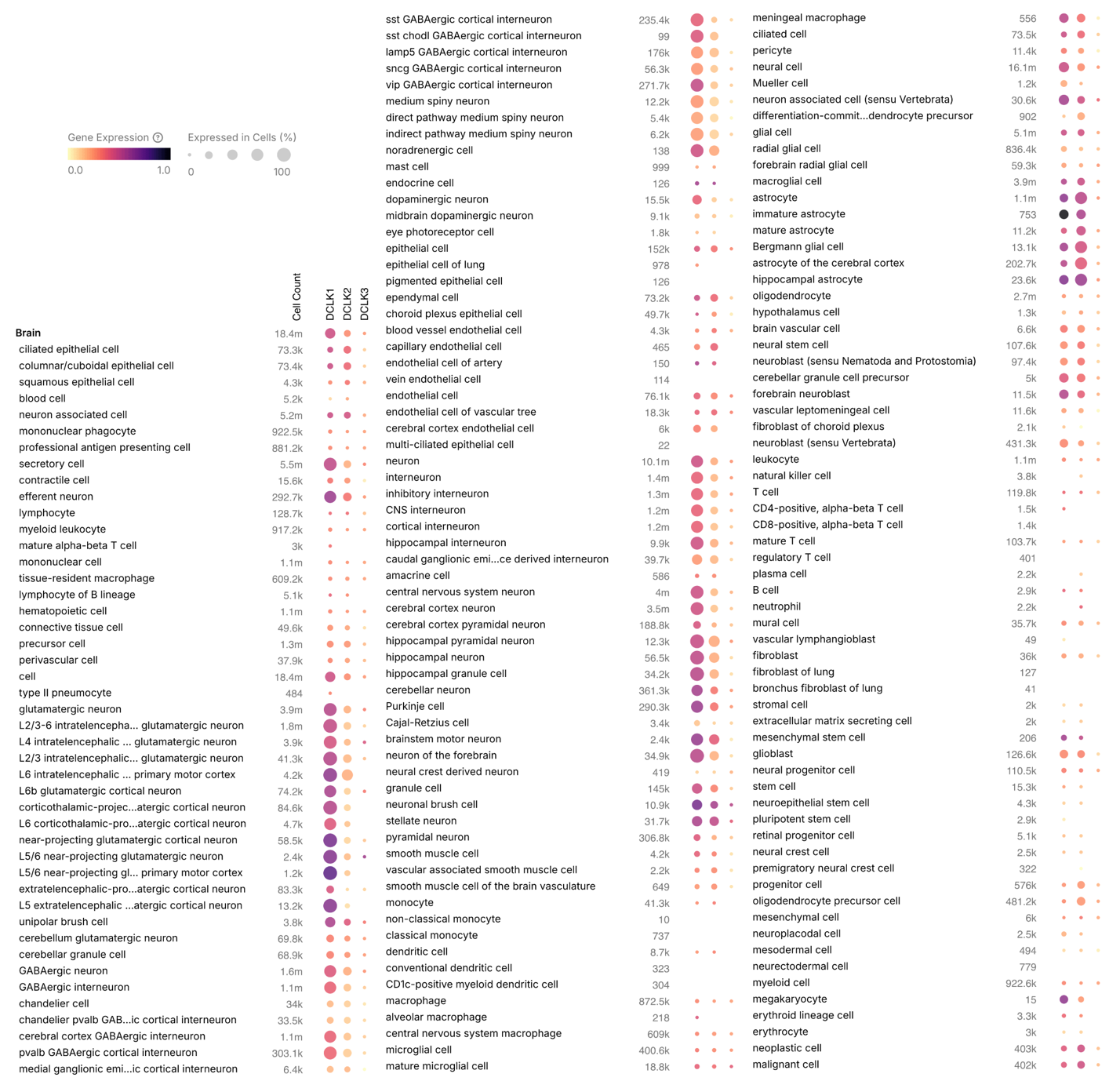
**Fig. S17.** DCLK expression in all human brain cell types deposited in CELLxGENE.
