# Supplementary material for: Deciphering the unique autoregulatory mechanisms and substrate specificity of the understudied DCLK3 kinase linked to neurodegenerative diseases
Source: J Biol Chem. 2025 Sep 1;301(10):110664. doi: 10.1016/j.jbc.2025.110664 (PMC12509982; doi:10.1016/j.jbc.2025.110664)
Supplement: Supporting Tables [file mmc2.docx]

**Supplementary Information for**

**Deciphering the unique autoregulatory mechanisms and substrate specificity of the understudied DCLK3 kinase linked to neurodegenerative diseases**

Jason D. Lu^1^, Peng Zhao^2^, Anup Prasad^1^, Neha Gupta^3^, Nathan Gravel^3^, Tej P. Shidhaye^1^, Lance Wells^1,2^, Samiksha Katiyar^3^* and Natarajan Kannan^1,3^*

^1^Department of Biochemistry and Molecular Biology, University of Georgia, Athens, GA 30602

^2^Complex Carbohydrate Research Center, University of Georgia, Athens, GA 30602

^3^Institute of Bioinformatics, University of Georgia, Athens, GA 30602

*Corresponding authors: Samiksha Katiyar ([samiksha@uga.edu](mailto:samiksha@uga.edu)) and Natarajan Kannan ([nkannan@uga.edu](mailto:nkannan@uga.edu))

**This file includes:**

Tables S1 to S4

| **Gene name** | **Uniprot accession** | **Phosphosite** |
| --- | --- | --- |
| **CCDC88B** | A6NC98 | S597 |
| **KIF1C** | O43896 | S1033 |
| **GLI1** | P08151 | S560 |
| **MAPT (Tau)** | P10636 | S531 |
| **KIF23** | Q02241 | S912 |
| **DST** | Q03001 | S3968 |
| **DST** | Q03001 | S7510 |
| **KIF19** | Q2TAC6 | S895 |
| **KIF21A** | Q7Z4S6 | S853 |
| **LUZP1** | Q86V48 | S574 |
| **NAV3** | Q8IVL0 | S1113 |
| **JAKMIP1** | Q96N16 | S382 |
| **RMDN3** | Q96TC7 | S46 |
| **GAS2L1** | Q99501 | S352 |
| **CAMSAP3** | Q9P1Y5 | S1074 |
| **MACF1** | Q9UPN3 | S3927 |
| **MACF1** | Q9UPN3 | S7330 |
| **MAST1** | Q9Y2H9 | S161 |
| **MTCL1** | Q9Y4B5 | S1814 |

**Table S1.** Phosformer-ST predicted interactors with score > 0.98 and association with the GO term “microtubule binding”. Cytoscape network shown in Figure S7.

|  | **Peptide** | **TAU_position** | **Phosformer-ST_score_DCLK3** | **Phosformer-ST_score_DCLK1** | **Phosformer-ST_score_DCLK2** |
| --- | --- | --- | --- | --- | --- |
| **0** | GSRSRTPSLPTPPTR | 214 | 0.9871106 | 0.9865616 | 0.9883609 |
| **1** | DTSPRHLSNVSSTGS | 409 | 0.843729 | 0.8285546 | 0.8249095 |
| **2** | RTPPKSPSSAKSRLQ | 237 | 0.81406903 | 0.8427994 | 0.87433213 |
| **3** | **KVTSKCGSLGNIHHK** | 324 | 0.7638957 | 0.74378467 | 0.7834074 |
| **4** | TPGSRSRTPSLPTPP | 212 | 0.67885923 | 0.67949605 | 0.7201753 |
| **5** | SAKSRLQTAPVPMPD | 245 | 0.29621407 | 0.36768335 | 0.41623205 |
| **6** | **DFKDRVQSKIGSLDN** | 352 | 0.22138824 | 0.2850099 | 0.3354917 |
| **7** | **VDLSKVTSKCGSLGN** | 320 | 0.17602913 | 0.24675927 | 0.3281316 |
| **8** | **RVQSKIGSLDNITHV** | 356 | 0.17548001 | 0.23549263 | 0.3679691 |
| **9** | **NVKSKIGSTENLKHQ** | 262 | 0.14608732 | 0.28496587 | 0.36282822 |
| **10** | PGTPGSRSRTPSLPT | 210 | 0.10673177 | 0.07614774 | 0.18016528 |
| **11** | **PDLKNVKSKIGSTEN** | 258 | 0.10232275 | 0.17071392 | 0.25556388 |
| **12** | NVQSKCGSKDNIKHV | 293 | 0.09675524 | 0.14577556 | 0.1466705 |
| **13** | VTQARMVSKSKDGTG | 129 | 0.08689012 | 0.096221074 | 0.10842897 |
| **14** | RENAKAKTDHGAEIV | 386 | 0.080153495 | 0.080838755 | 0.11584521 |
| **15** | KSPSSAKSRLQTAPV | 241 | 0.06657082 | 0.08157579 | 0.11493048 |
| **16** | KIETHKLTFRENAKA | 377 | 0.053694755 | 0.08324145 | 0.09130029 |
| **17** | GSPGTPGSRSRTPSL | 208 | 0.028742984 | 0.10675355 | 0.11005673 |
| **18** | PAPKTPPSSGEPPKS | 184 | 0.012353844 | 0.017367786 | 0.018986437 |
| **19** | LDLSNVQSKCGSKDN | 289 | 0.011346306 | 0.018104231 | 0.029602632 |
| **20** | PPKSGDRSGYSSPGS | 195 | 0.010625548 | 0.040677674 | 0.060778596 |
| **21** | GGNKKIETHKLTFRE | 373 | 0.0067319497 | 0.013101194 | 0.015899064 |
| **22** | VSKSKDGTGSDDKKA | 135 | 0.0065308753 | 0.03192487 | 0.070857 |
| **23** | SSGEPPKSGDRSGYS | 191 | 0.005080201 | 0.0027483096 | 0.008808185 |
| **24** | DEAAGHVTQARMVSK | 123 | 0.0048935604 | 0.012219443 | 0.013897143 |
| **25** | QARMVSKSKDGTGSD | 131 | 0.003654827 | 0.0029906798 | 0.0051814234 |
| **26** | SGDRSGYSSPGSPGT | 198 | 0.003172698 | 0.0040479423 | 0.009804557 |
| **27** | KHVPGGGSVQIVYKP | 305 | 0.0031401408 | 0.009543656 | 0.005175874 |
| **28** | TPPKSPSSAKSRLQT | 238 | 0.0026255634 | 0.0040877457 | 0.0057296488 |
| **29** | PRHLSNVSSTGSIDM | 412 | 0.0025956575 | 0.0036581843 | 0.0041043824 |
| **30** | RHLSNVSSTGSIDMV | 413 | 0.0024946863 | 0.008990763 | 0.0077608153 |
| **31** | SDAKSTPTAEDVTAP | 71 | 0.002406692 | 0.008359067 | 0.00822121 |
| **32** | ATRIPAKTPPAPKTP | 175 | 0.00232128 | 0.0033659486 | 0.0042664246 |
| **33** | SNVSSTGSIDMVDSP | 416 | 0.0018309864 | 0.0041366746 | 0.00385298 |
| **34** | APKTPPSSGEPPKSG | 185 | 0.0016335013 | 0.001600667 | 0.0030361386 |
| **35** | DGKTKIATPRGAAPP | 153 | 0.0015884391 | 0.0022016985 | 0.002670696 |
| **36** | GGQVEVKSEKLDFKD | 341 | 0.0014921238 | 0.0018840574 | 0.003058285 |
| **37** | INKKLDLSNVQSKCG | 285 | 0.0012967539 | 0.0018756489 | 0.0025265901 |
| **38** | VVRTPPKSPSSAKSR | 235 | 0.00124007 | 0.001769187 | 0.0016179197 |
| **39** | GDRSGYSSPGSPGTP | 199 | 0.0011398438 | 0.0017794532 | 0.0015664683 |
| **40** | PVDLSKVTSKCGSLG | 319 | 0.0010659847 | 0.0008662252 | 0.00097577897 |
| **41** | AKGADGKTKIATPRG | 149 | 0.0010077889 | 0.001610408 | 0.003969986 |
| **42** | AGIGDTPSLEDEAAG | 113 | 0.000789313 | 0.0010611193 | 0.0013596837 |
| **43** | SSPGSPGTPGSRSRT | 205 | 0.0007800275 | 0.0009021265 | 0.0006650118 |
| **44** | QKGQANATRIPAKTP | 169 | 0.0007646813 | 0.0010440298 | 0.0011899049 |
| **45** | PSLPTPPTREPKKVA | 220 | 0.000706482 | 0.0009531253 | 0.0010886198 |
| **46** | VKSKIGSTENLKHQP | 263 | 0.00070283585 | 0.0007450911 | 0.0006696385 |
| **47** | RKDQGGYTMHQDQEG | 30 | 0.0007016524 | 0.0011745107 | 0.0018807102 |
| **48** | KKVAVVRTPPKSPSS | 231 | 0.00066789036 | 0.00065107644 | 0.0005405832 |
| **49** | VYKPVDLSKVTSKCG | 316 | 0.00059181784 | 0.0007655144 | 0.00092916976 |
| **50** | VYKSPVVSGDTSPRH | 400 | 0.00054728857 | 0.0008418943 | 0.00102053 |
| **51** | SGYSSPGSPGTPGSR | 202 | 0.000520494 | 0.00068293395 | 0.00055639324 |
| **52** | ESPLQTPTEDGSEEP | 52 | 0.00051727245 | 0.00074031507 | 0.0009370664 |
| **53** | KTPPAPKTPPSSGEP | 181 | 0.0005041982 | 0.00054978626 | 0.0004635511 |
| **54** | SPVVSGDTSPRHLSN | 403 | 0.00049879117 | 0.0005690874 | 0.0007428749 |
| **55** | SETSDAKSTPTAEDV | 68 | 0.0004810753 | 0.00053692923 | 0.0006173919 |
| **56** | PVVSGDTSPRHLSNV | 404 | 0.00047388594 | 0.00075117755 | 0.0008359581 |
| **57** | KSKDGTGSDDKKAKG | 137 | 0.00047180898 | 0.0007623448 | 0.00092276116 |
| **58** | TDAGLKESPLQTPTE | 46 | 0.00045480815 | 0.0003874791 | 0.0003747874 |
| **59** | VDSPQLATLADEVSA | 427 | 0.00042317944 | 0.0006323616 | 0.00085614296 |
| **60** | GAEIVYKSPVVSGDT | 396 | 0.00039520147 | 0.0003859363 | 0.00034020742 |
| **61** | ETSDAKSTPTAEDVT | 69 | 0.00036391118 | 0.00035647396 | 0.0003368141 |
| **62** | LADEVSASLAKQGL- | 435 | 0.00035118373 | 0.0005751876 | 0.00061789696 |
| **63** | SRTPSLPTPPTREPK | 217 | 0.0003366847 | 0.00036796907 | 0.00029406307 |
| **64** | VMEDHAGTYGLGDRK | 17 | 0.0003278042 | 0.00078176643 | 0.00064840505 |
| **65** | DGSEEPGSETSDAKS | 61 | 0.00031212508 | 0.0004011314 | 0.00036267133 |
| **66** | EEPGSETSDAKSTPT | 64 | 0.00028411217 | 0.00044922484 | 0.00047711632 |
| **67** | QAAAQPHTEIPEGTT | 95 | 0.00027768023 | 0.00033585183 | 0.0003968466 |
| **68** | QTPTEDGSEEPGSET | 56 | 0.00023814994 | 0.00024270307 | 0.0002466746 |
| **69** | HLSNVSSTGSIDMVD | 414 | 0.00021859401 | 0.00026670584 | 0.00024291466 |
| **70** | SEEPGSETSDAKSTP | 63 | 0.00021702319 | 0.00027517413 | 0.00026192624 |
| **71** | ATLADEVSASLAKQG | 433 | 0.00020984547 | 0.00027312888 | 0.00026274336 |
| **72** | EEAGIGDTPSLEDEA | 111 | 0.00019880006 | 0.0002062344 | 0.00018365547 |
| **73** | IGSLDNITHVPGGGN | 361 | 0.00019610111 | 0.0002339109 | 0.00022921542 |
| **74** | LKESPLQTPTEDGSE | 50 | 0.00019184426 | 0.00017839695 | 0.0001591008 |
| **75** | HQDQEGDTDAGLKES | 39 | 0.00018748593 | 0.00023884141 | 0.00022131696 |
| **76** | HTEIPEGTTAEEAGI | 101 | 0.00017192365 | 0.00020801077 | 0.0002003968 |
| **77** | GSIDMVDSPQLATLA | 422 | 0.00017131587 | 0.00021169837 | 0.00017250609 |
| **78** | TEIPEGTTAEEAGIG | 102 | 0.00016420988 | 0.00019727285 | 0.00017009041 |
| **79** | TPTAEDVTAPLVDEG | 76 | 0.00013965098 | 0.00015068073 | 0.0001331196 |

**Table S2.** Phosformer-ST predicted scores for DCLK1, DCLK2, and DCLK3 across all S/T 15-mer peptides in Tau. The peptides are sorted (from highest to lowest) based on phosphorylation scores for DCLK3. Peptides experimentally validated to be phosphorylated by DCLK3 kinase domain in our synthetic Tau construct are highlighted in bold.

| **UniProtID** | **Protein name** | **Sequence** | **Organism** | **Length (aa)** | **Modification/ligand notes (PTMs)** | **Truncation/domain notes** | **Used in** |
| --- | --- | --- | --- | --- | --- | --- | --- |
| Q8N568 | DCLK2 | YKIGKVIGDGNFAVVKECIDRSTGKEFALKIIDKAKCCGKEHLIENEVSILRRVKHPNIIMLVEEMETATELFLVMELVKGGDLFDAITSSTKYTERDGSAMVYNLANALRYLHGLSIVHRDIKPENLLVCEYPDGTKSLKLGDFGLATVVEGPLYTVCGTPTYVAPEIIAETGYGLKVDIWAAGVITYILLCGFPPFRSENNLQEDLFDQILAGKLEFPAPYWDNITDSAKELISQMLQVNVEARCTAGQILSHPWVSDDASQENNMQAEVTGKLKQHFNNALPKQNSTTTGVSVIMNTALDKEGQIFCSKHCQDSGRPGMEPISPVPPSVEEIPVPGEAVPAPTPPESPTPHPPPAAPGGERAGTWRRHRD | Homo sapiens | 373 | none | Kinase domain + C-tail (residues 394-766) | DCLK2 (Fig. 1B, Fig. S1) |
| Q9C098 | DCLK3 | YETGRVIGDGNFAVVKECRHRETRQAYAMKIIDKSRLKGKEDMVDSEILIIQSLSHPNIVKLHEVYETDMEIYLILEYVQGGDLFDAIIESVKFPEPDAALMIMDLCKALVHMHDKSIVHRDLKPENLLVQRNEDKSTTLKLADFGLAKHVVRPIFTVCGTPTYVAPEILSEKGYGLEVDMWAAGVILYILLCGFPPFRSPERDQDELFNIIQLGHFEFLPPYWDNISDAAKDLVSRLLVVDPKKRYTAHQVLQHPWIETAGKTNTVKRQKQVSPSSEGHFRSQHKRVVEQVS | Homo sapiens | 293 | none | Kinase domain + C-tail (residues 356-648) | DCLK3 (Fig. 1B, Fig. S1, Fig. S6) |
| Q9C098 | DCLK3 | YETGRVIGDGNFAVVKECRHRETRQAYAMKIIDKSRLKGKEDMVDSEILIIQSLSHPNIVKLHEVYETDMEIYLILEYVQGGDLFDAIIESVKFPEPDAALMIMDLCKALVHMHDKSIVHRDLKPENLLVQRNEDKSTTLKLADFGLAKHVVRPIFTVCGTPTYVAPEILSEKGYGLEVDMWAAGVILYILLCGFPPFRSPERDQDELFNIIQLGHFEFLPPYWDNISDAAKDLVSRLLVVDPKKRYTAHQVLQHPWIETAGKTNTVKRQKQVSPSSEGHFRSQHKRVVEQVS | Homo sapiens | 293 | pT358, pT619, pS631, pS632, pS638 | Kinase domain + C-tail (residues 356-648) | pDCLK3  (Fig. 3B, 3D, 3F, 3G, Fig. S1, Fig. S6) |
| Q9C098 | DCLK3 | MGKEPLTLKSIQVAVEELYPNKARALTLAQHSRAPSPRLRSRLFSKALKGDHRCGETETPKSCSEVAGCKAAMRHQGKIPEELSLDDRARTQKKWGRGKWEPEPSSKPPREATLEERHARGEKHLGVEIEKTSGEIIRCEKCKRERELQQSLERERLSLGTSELDMGKGPMYDVEKLVRTRSCRRSPEANPASGEEGWKGDSHRSSPRNPTQELRRPSKSMDKKEDRGPEDQESHAQGAAKAKKDLVEVLPVTEEGLREVKKDTRPMSRSKHGGWLLREHQAGFEKLRRTRGEEKEAEKEKKPCMSGGRRMTLRDDQPAKLEKEPKTRPEENKPERPSGRKPRPMGIIAANVEKHYETGRVIGDGNFAVVKECRHRETRQAYAMKIIDKSRLKGKEDMVDSEILIIQSLSHPNIVKLHEVYETDMEIYLILEYVQGGDLFDAIIESVKFPEPDAALMIMDLCKALVHMHDKSIVHRDLKPENLLVQRNEDKSTTLKLADFGLAKHVVRPIFTVCGTPTYVAPEILSEKGYGLEVDMWAAGVILYILLCGFPPFRSPERDQDELFNIIQLGHFEFLPPYWDNISDAAKDLVSRLLVVDPKKRYTAHQVLQHPWIETAGKTNTVKRQKQVSPSSEGHFRSQHKRVVEQVS | Homo sapiens | 648 | none | Full-length | DCLK3 (Fig. 6B and 6C, Fig. S1) |
| P10636-8 | Tau-F  (2N4R) | MAEPRQEFEVMEDHAGTYGLGDRKDQGGYTMHQDQEGDTDAGLKESPLQTPTEDGSEEPGSETSDAKSTPTAEDVTAPLVDEGAPGKQAAAQPHTEIPEGTTAEEAGIGDTPSLEDEAAGHVTQARMVSKSKDGTGSDDKKAKGADGKTKIATPRGAAPPGQKGQANATRIPAKTPPAPKTPPSSGEPPKSGDRSGYSSPGSPGTPGSRSRTPSLPTPPTREPKKVAVVRTPPKSPSSAKSRLQTAPVPMPDLKNVKSKIGSTENLKHQPGGGKVQIINKKLDLSNVQSKCGSKDNIKHVPGGGSVQIVYKPVDLSKVTSKCGSLGNIHHKPGGGQVEVKSEKLDFKDRVQSKIGSLDNITHVPGGGNKKIETHKLTFRENAKAKTDHGAEIVYKSPVVSGDTSPRHLSNVSSTGSIDMVDSPQLATLADEVSASLAKQGL | Homo sapiens | 441 | none | Full-length | Tau 2N4R (Fig. 4A) |

**Table S3.** Input sequences and experimental constructs

| **Model** | **Phosphosite** | **Total atoms** | **Box dimension (Å)** | **Simulation time (ns)** |
| --- | --- | --- | --- | --- |
| DCLK3 | No | 95939 | 98.3, 98.3, 98.3 | 500 |
| pDCLK3 | T358, T619, S631, S632, S638 | 95921 | 98.3, 98.3, 98.3 | 500 |

**Table S4.** Molecular dynamics simulation details.
